# Clinical Phenotype and Outcome of Hypertrophic Cardiomyopathy Associated With Thin-Filament Gene Mutations

**DOI:** 10.1016/j.jacc.2014.09.059

**Published:** 2014-12-23

**Authors:** Raffaele Coppini, Carolyn Y. Ho, Euan Ashley, Sharlene Day, Cecilia Ferrantini, Francesca Girolami, Benedetta Tomberli, Sara Bardi, Francesca Torricelli, Franco Cecchi, Alessandro Mugelli, Corrado Poggesi, Jil Tardiff, Iacopo Olivotto

**Affiliations:** ∗ Center of Molecular Medicine (CIMMBA), University of Florence, Florence, Italy; † Brigham and Women’s Hospital, Boston, Massachusetts; ‡ Cardiovascular Medicine, Stanford Medical Center, Stanford, California; § University of Michigan Medical Center, Ann Arbor, Michigan; ‖ Genetics Unit; Careggi University Hospital, Florence, Italy; ¶ Referral Center for Cardiomyopathies, Careggi University Hospital, Florence, Italy; # Department of Cellular and Molecular Medicine, University of Arizona, Tucson, Arizona

**Keywords:** diastolic function, end-stage, genotype to phenotype correlation, triphasic filling, troponin, *ACTC*, cardiac α-actin gene, AF, atrial fibrillation, CMR, cardiac magnetic resonance, ECG, electrocardiography, HCM, hypertrophic cardiomyopathy, HR, hazard ratio, ICD, implantable cardioverter-defibrillator, LGE, late gadolinium enhancement, LV, left ventricular, LVH, left ventricular hypertrophy, *MYBPC3*, myosin binding protein C, *MYH7*, myosin heavy chain, NSVT, nonsustained ventricular tachycardia, NYHA, New York Heart Association, SCD, sudden cardiac death, *TNNT2*, cardiac troponin T gene, *TNNI3*, cardiac troponin I gene, *TPM1*, cardiac α-tropomyosin gene

## Abstract

**Background:**

Mild hypertrophy but increased arrhythmic risk characterizes the stereotypic phenotype proposed for hypertrophic cardiomyopathy (HCM) caused by thin-filament mutations. However, whether such clinical profile is different from more prevalent thick-filament–associated disease is unresolved.

**Objectives:**

This study aimed to assess clinical features and outcomes in a large cohort of patients with HCM associated with thin-filament mutations compared with thick-filament HCM.

**Methods:**

Adult HCM patients (age >18 years), 80 with thin-filament and 150 with thick-filament mutations, were followed for an average of 4.5 years.

**Results:**

Compared with thick-filament HCM, patients with thin-filament mutations showed: 1) milder and atypically distributed left ventricular (LV) hypertrophy (maximal wall thickness 18 ± 5 mm vs. 24 ± 6 mm; p < 0.001) and less prevalent outflow tract obstruction (19% vs. 34%; p = 0.015); 2) higher rate of progression to New York Heart Association functional class III or IV (15% vs. 5%; p = 0.013); 3) higher prevalence of systolic dysfunction or restrictive LV filling at last evaluation (20% vs. 9%; p = 0.038); 4) 2.4-fold increase in prevalence of triphasic LV filling pattern (26% vs. 11%; p = 0.002); and 5) similar rates of malignant ventricular arrhythmias and sudden cardiac death (p = 0.593).

**Conclusions:**

In adult HCM patients, thin-filament mutations are associated with increased likelihood of advanced LV dysfunction and heart failure compared with thick-filament disease, whereas arrhythmic risk in both subsets is comparable. Triphasic LV filling is particularly common in thin-filament HCM, reflecting profound diastolic dysfunction.

Hypertrophic cardiomyopathy (HCM) is the most common genetic heart disease, generally caused by mutations in cardiac sarcomere genes (1). Most genotyped HCM patients harbor defects in the thick-filament genes, myosin heavy chain (*MYH7*) and myosin binding protein C (*MYBPC3*) (2). However, in a distinct patient subgroup, the disease is caused by mutations in thin-filament genes, including cardiac troponin T (*TNNT2*) and I (*TNNI3*), α-tropomyosin (*TPM1*), and cardiac actin (*ACTC*) 3, 4. Each accounts for a small proportion of HCM cohorts, with *TNNT2*, the most common, accounting for only 2% to 5% (5).

The thin filament is a multisubunit, allosterically regulated molecular machine (Figure 1); thus, mutations in any of its components should exert similar biophysical effects and pathophysiological consequences 4, 6. However, a comprehensive assessment of the clinical presentation and outcome of patients carrying thin-filament mutations, compared with thick-filament disease, has not yet been performed. Initial clinical phenotype descriptions of *TNNT2* and *TNNI3* mutations were from families with severe HCM, characterized by high incidence of sudden cardiac death (SCD) despite relatively mild hypertrophy, often in children and adolescents 7, 8, 9, 10. Identification of mutations in these genes is therefore potentially relevant to clinical decision-making, including risk stratification for arrhythmic prophylaxis. However, subsequent reports of larger, less-selected cohorts show wide phenotypic and clinical variability for individual thin-filament genes, similar to thick-filament HCM 5, 11, 12. Consequently, whether thin-filament HCM has a truly distinct clinical profile from thick-filament HCM is unresolved. This study specifically addressed this issue by evaluating the clinical spectrum, echocardiographic features, and outcomes of a large, multicenter, genotyped cohort with HCM.

**Figure 1:**
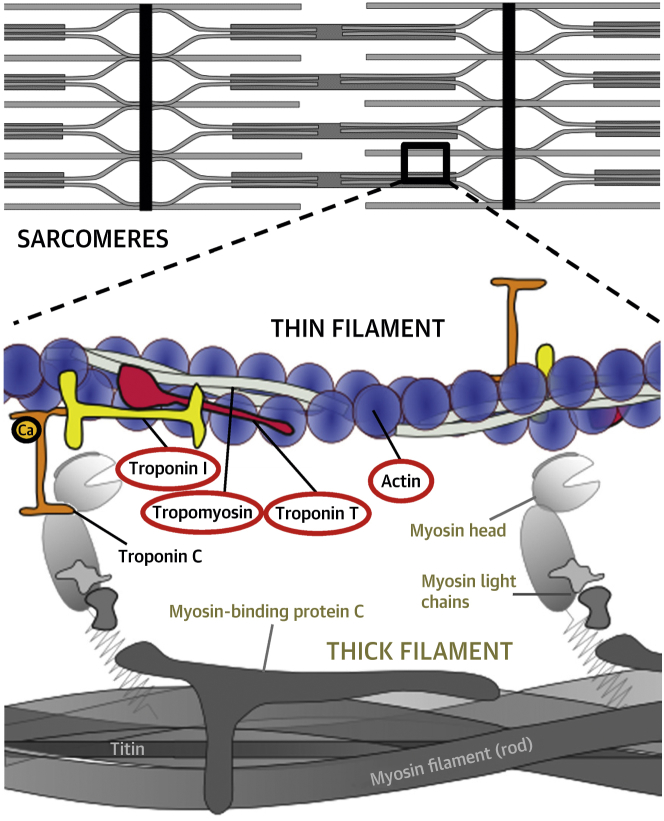
The Cardiac Sarcomere Thin Filament Schematic representation of the thin filament and its key molecular components **(colored)** in relation to thick-filament proteins **(gray)**. Thin-filament proteins with disease-causing mutations found in the thin-filament cohort of this study are circled in **red**.

## Methods

### Patient population

All participants were unrelated index patients. HCM diagnosis was by 2-dimensional echocardiographic identification of a hypertrophied (≥13 mm), nondilated LV, in the absence of another cardiac or systemic disease capable of producing that magnitude of ventricular hypertrophy (13). The study included 80 HCM patients (8% of HCM patients genotyped during this time) with a pathogenic or likely pathogenic cardiac thin-filament gene mutation identified between January 2001 and December 2009 at 4 referral centers: Careggi University Hospital, Florence, Italy; Brigham and Women’s Hospital, Boston, Massachusetts; Stanford Medical Center, Palo Alto, California, and the University of Michigan Medical Center, Ann Arbor, Michigan (Table 1).

**Table 1:** Baseline Clinical Features

	Thin Filament (n = 80)	Thick Filament (n = 150)	p Value
Clinical/demographic features			
Female	36 (45)	66 (44)	0.488
Age at enrollment, yrs	44 ± 16	42 ± 17	0.387
Age at final evaluation, yrs	49 ± 16	47 ± 17	0.388
Family history of HCM	35 (44)	67 (44)	0.497
Family history of sudden cardiac death	29 (36)	28 (18)	0.004
NYHA functional class			
I	53 (66)	92 (61)	0.227
II	21 (26)	43 (29)	0.375
III/IV	6 (8)	16 (10)	0.613
Angina pectoris	16 (20)	30 (20)	0.512
Syncope	14 (18)	21 (14)	0.545
Symptomatic	43 (54)	75 (50)	0.588
Atrial fibrillation	25 (31)	49 (30)	0.827
Abnormal BP response to exercise	19 (24)	19 (13)	0.037
Nonsustained ventricular tachycardia	24 (30)	26 (17)	0.032
Sustained ventricular tachycardia	6 (8)	8 (5)	0.395
ECG			
T-wave inversion	54 (67)	66 (44)	0.002
Increased voltage (LV hypertrophy)	48 (60)	97 (65)	0.514
Inferolateral Q waves	30 (37)	14 (9)	<0.001
LV strain/repolarization abnormalities	37 (46)	51 (34)	0.087
Echocardiography			
Left atrial diameter, mm	44 ± 8	43 ± 8	0.367
Maximum LV wall thickness, mm	18 ± 5	24 ± 6	<0.001
With LV wall thickness >30 mm	6 (7)	26 (17)	0.028
Maximal thickness site			
Septum	55 (69)	141 (94)	<0.001
Apex	16 (20)	7 (5)	<0.001
Concentric	9 (11)	2 (1)	<0.001
LV end-diastolic diameter, mm	44 ± 7	45 ± 7	0.303
LV end-systolic diameter, mm	28 ± 7	27 ± 8	0.347
LV ejection fraction, %	65 ± 10	68 ± 12	0.057
With LV ejection fraction <50%	4 (5)	8 (5)	0.420
LVOT gradient, mm Hg	15 ± 24	24 ± 24	0.007
LVOT obstruction	15 (19)	51 (34)	0.015
Moderate-to-severe mitral regurgitation	5 (6)	13 (9)	0.309
LV filling pattern			
Normal	24 (32)	44 (35)	0.459
Impaired relaxation	26 (35)	43 (25)	0.176
Pseudonormalized	18 (25)	35 (28)	0.380
Restrictive	6 (8)	3 (2)	0.064
Triphasic LV filling	21 (26)	14 (11)	0.002
Lateral E′, cm/s	8.1 ± 3.3	10.6 ± 3.6	<0.001
Cardiac magnetic resonance			
Study performed	47 (59)	76 (51)	0.268
LV ejection fraction, %	65 ± 11	71 ± 11	0.004
LV mass index, g/m^2^	87 ± 27	99 ± 38	0.013
LGE present	40 (85)	61 (80)	0.630
LGE extent, % of LV mass	20 ± 11	16 ± 8	0.002
LGE >30% of LV mass	12 (27)	8 (11)	0.042

Values are n (%) or mean ± SD.

BP = blood pressure; ECG = electrocardiograph; HCM = hypertrophic cardiomyopathy; LGE = late gadolinium enhancement; LV = left ventricular; LVOT= left ventricular outflow tract; NYHA = New York Heart Association.

For comparison, we evaluated 150 HCM patients with pathogenic or likely pathogenic mutations in the cardiac thick-filament genes *MYH7* and *MYBPC3* and the regulatory light chain (*MYL2*) consecutively identified in Florence during the same period. Clinical features of this reference group (Table 1) closely recapitulate published HCM cohorts from Europe and the United States 14, 15, 16, largely comprising thick-filament patients. Previous collaborative studies excluded significant discrepancy between cohorts from Florence and other centers 14, 17.

### Mutational analysis

After informed consent, patients were screened for mutations in protein-coding exons and splice sites of 8 myofilament genes, including the thin-filament genes *TNNT2*, *TNNI3*, *TPM1*, and *ACTC*; the thick-filament genes *MYBPC3*, *MYH7*, *MYL2*; and the essential light chain (*MYL3*). Genetic testing using established methods available at screening was performed by Clinical Laboratory Improvement Amendments (CLIA)–certified laboratories in the United States and at the Genetics Unit of Careggi University Hospital in Florence (2). Direct Sanger sequencing confirmed every variant. Variants were considered pathogenic if published as causative HCM mutations in at least 2 independent peer-reviewed studies. Novel mutations fulfilling the following internationally recommended criteria were considered likely to be pathogenic (18): 1) nonsynonymous variant causing an amino acid change in a residue highly conserved among species and predicted to significantly damage protein structure or function (Grantham, SIFT, and Polyphen scores), or truncating mutation; 2) the variant was absent in healthy control populations, including filtering for 1000 Genomes Project, National Heart, Lung and Blood Institute Exome Sequencing Project, and the Single Nucleotide Polymorphism database with minimal allelic frequency of <0.05; and 3) cosegregation with affected family members could be demonstrated for at least 1 patient. Before patient enrollment, the attending cardiologist and clinical geneticists evaluated this information on a case-by-case basis to confirm variant interpretation. Details of mutation distribution and classification in the thin- and thick-filament cohorts are found in Online Tables 1 and 2, respectively. At the beginning of the study, available published information was used to classify variant pathogenicity. Recent next-generation sequencing data led to subsequent downgrading of 3 thin-filament variants from likely pathogenic to variants of uncertain significance (*TNNT2*-Arg278Cys, *TNNT2*-Asn262Ser, and *TNNI3*-Arg162Pro). All remain potential disease-causing candidates, and there is evidence of Arg278Cys cosegregation in our cohort; thus these mutations were included in the analysis.

To avoid bias related to founder effects, only the first identified patient carrying each of 2 highly recurrent mutations (i.e., E258K [Glu258Lys] in *MYBPC3* and R869H [Arg869His] in *MYH7* present in 52 and 19 Florence index patients, respectively) were included (2). Patients with complex genotypes, including thin-filament mutations associated with pathogenic or likely pathogenic *MYBPC3* or *MHY7* variants, were excluded.

### Echocardiography

Echocardiographic studies were performed as described (14) using commercially available instruments. LV filling patterns were assessed by pulsed-wave Doppler at the mitral tip level, and combined with tissue-Doppler evaluation of lateral mitral annulus velocity. We identified 4 LV filling patterns: (1 = normal; 2 = abnormal relaxation; 3 = pseudonormal; 4 = restrictive), defined according to existing guidelines 19, 20. Triphasic LV filling was considered present when a velocity peak of at least 0.2 m/s (an L-wave) was seen during diastasis (21), independent of the overall LV filling pattern.

### Cardiac magnetic resonance

Cardiac magnetic resonance (CMR) imaging, including evaluation of late gadolinium enhancement (LGE), was performed as described (22) in a subset of patients using commercially available 1.5-T scanners.

### Follow-up and clinical outcomes

Patients were followed up at yearly intervals or more often if clinically indicated, with review of history and symptoms, physical examination, echocardiographic examination, and 12-lead electrocardiography (ECG). If clinically indicated, ambulatory ECG monitoring for 24 to 48 h and CMR were performed. Established risk factors for SCD were defined as prior cardiac arrest or sustained ventricular tachycardia; family history of SCD at ≤40 years of age; nonvasovagal syncope; multiple episodes of nonsustained ventricular tachycardia (NSVT) during repeated ambulatory ECGs; maximal LV wall thickness ≥30 mm; and abnormal blood pressure response to exercise 13, 23.

We documented major clinical outcomes including cardiovascular death, resuscitated cardiac arrest, nonfatal stroke, and progression to severe congestive symptoms (New York Heart Association [NYHA] functional class III or IV). Advanced LV dysfunction was defined by echocardiographic detection of systolic impairment with an ejection fraction <50% (generally used to identify end-stage HCM [24]) and/or restrictive LV filling pattern.

### Statistical analysis

Unpaired Student *t* tests were used to compare normally distributed data from patients with thick- and thin-filament HCM. Chi-square or Fisher exact tests were used to compare noncontinuous variables expressed as proportions. Survival curves were constructed according to the Kaplan-Meier method, and comparisons were performed using the log-rank test. Cox proportional hazard models were used to assess the effects of multiple clinical features on the risk of outcome events and to estimate survival curves of thin- versus thick-filament patients at net of covariates. Time of first clinical evaluation at each institution was considered as time 0. The probability values are 2-sided and considered significant when <0.05. Calculations were performed using SPSS version 20.0 software (SPSS, Chicago, Illinois).

## Results

### Genetic profile of thin-filament HCM

**Figure 2:**
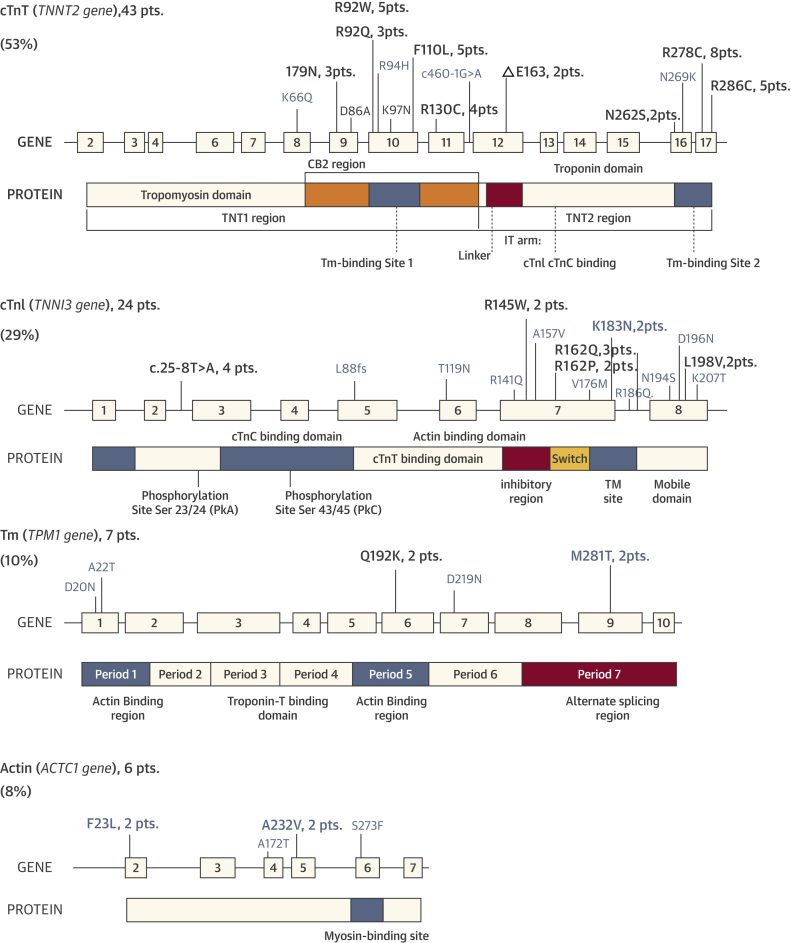
Genetic Basis of Thin-Filament HCM Exons and structural and functional domains of cardiac troponin T (cTnT) and I (cTnI), tropomyosin (Tm), and actin, showing mutation sites identified in the study cohort. **Black**, certainly pathogenic mutations (see Methods); **slate-grey**, mutations likely to be pathogenic. For mutations carried by more than 1 patient, the total number of carriers is indicated. HCM = hypertrophic cardiomyopathy.

A total of 39 different pathogenic or likely pathogenic sequence variants were identified in 80 unrelated probands with thin-filament HCM (Figure 2, Online Table 1), including 35 missense, 2 truncation, and 2 insertion/deletion mutations. *TNNT2* defects were the most common, with 15 distinct mutations identified in 43 of the 80 patients (53%). Additionally, we identified 15 different *TNNI3* mutations in 24 patients (30%), 5 *TPM1* mutations in 7 patients (9%), and 4 *ACTC* mutations in 6 patients (8%). None carried double thin-filament mutations. Patients carrying mutations in the most represented genes, *TNNT2* and *TNNI3*, showed remarkably similar clinical features and outcome profiles (Online Tables 3 and 4). Among the 150 thick-filament HCM patients enrolled for comparison (age 42 ± 17 years, 44% female), 94 different mutations were identified (Online Table 2), including 49 in *MYBPC3* (n = 83, 55%), 40 in *MHY7* (n = 57, 38%), and 5 in *MYL2* (n = 10, 7%).

### Baseline clinical characteristics of thin- versus thick-filament HCM

#### Clinical status

At initial evaluation, the mean age of the 80 thin-filament HCM patients was 44 ± 16 years; 45% were women. Most (66%) reported normal exercise tolerance (NYHA functional class I); however, 54% were symptomatic as a result of atrial fibrillation (AF) (30%), angina (20%), or syncope (18%) (Table 1). Overall, these features were comparable to the thick-filament cohort (Table 1). On 12-lead ECG, 37% of thin-filament patients showed inferolateral Q waves (vs. 9% in the 150 thick-filament patients, p < 0.001) and 67% showed inverted T waves in the precordial leads (vs. 44% of thick-filament patients, p = 0.002) (Table 1).

#### Cardiac imaging

**Figure 3:**
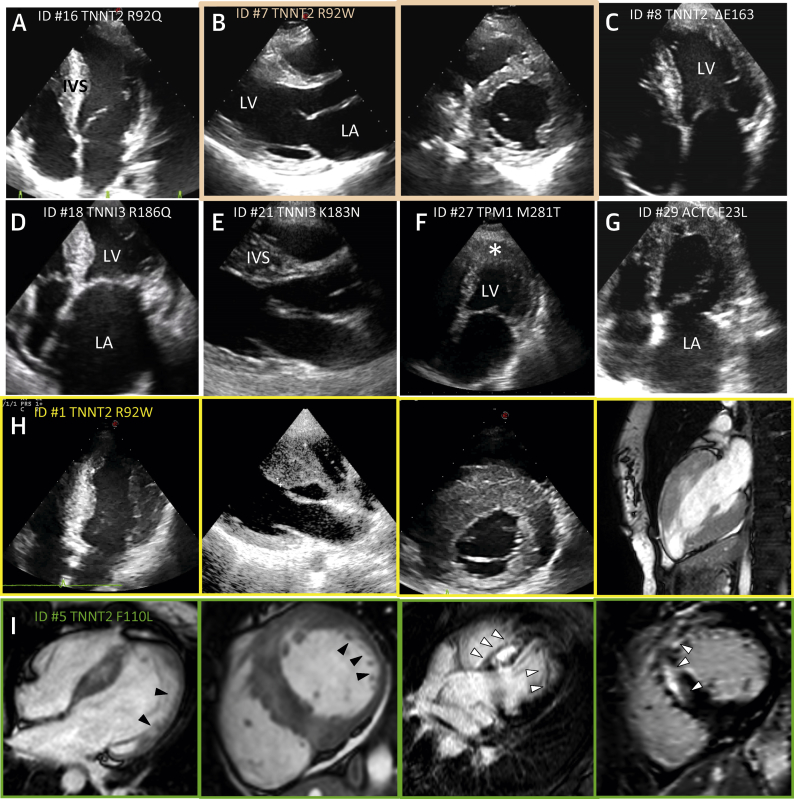
Phenotypic Variability in Thin-Filament–Associated HCM Echocardiographic and cardiomagnetic resonance (CMR) images showing variable extent and distribution of left ventricular hypertrophy (LVH) in patients from the thin-filament study cohort (patient’s ID number and mutation are indicated). **(A)** Moderate LVH associated with TNNT2-R92Q, with classic localization at the interventricular septum (IVS). **(B)** Mild midseptal hypertrophy and marked left atrial (LA) dilation associated with TNNT2-ΔE163. **(C)** Mild concentric LVH associated with TNNT2-R92W. **(D)** Moderate midseptal LVH with small LV cavity size and severe LA dilation (restrictive evolution) associated with TNNI3-R186Q. **(E)** Classic asymmetric septal LVH associated with TNNI3-K183M. **(F)** Apical LVH associated with TPM-M281T; **∗**indicates LV apex. **(G)** Apical LVH with severe LA dilation associated with ACTC-F23L. **(H)** Markedly asymmetric LVH involving the anterior and posterior septum and anterior wall associated with TNNT2-R92W. **(I)** CMR images from a patient carrying the TNNT2-F110L mutation. **(From left)** Four-chamber and midventricular short-axis views showing extensive regions of noncompaction **(black arrowheads)** in the LV apical and free wall regions; 4-chamber and short-axis views showing large areas of late gadolinium enhancement **(white arrowheads)** in the anterior free wall and in the septum, respectively. HCM = hypertrophic cardiomyopathy.

Several differences between thick- and thin-filament HCM in LV morphology and function were noted. Patients with thin-filament mutations had lesser maximal LV wall thickness values than the thick-filament group (18 ± 5 mm vs. 24 ± 6 mm; p < 0.001) and more often exhibited atypically distributed hypertrophy (31%), including concentric and apical patterns, whereas 94% of thick-filament HCM presented as classic asymmetric LVH involving the basal septum and anterior wall (p < 0.01) (Table 1, Figure 3), consistent with the lower prevalence of resting LV outflow tract obstruction in thin-filament patients (19% vs. 34% in thick-filament HCM, p = 0.015). An apical or concentric distribution of hypertrophy was most likely in *TNNI3* patients (41%) (Online Table 3). Aspects of LV noncompaction were uncommon; for example, none of the 6 patients with actin mutations showed regional noncompaction.

**Figure 4:**
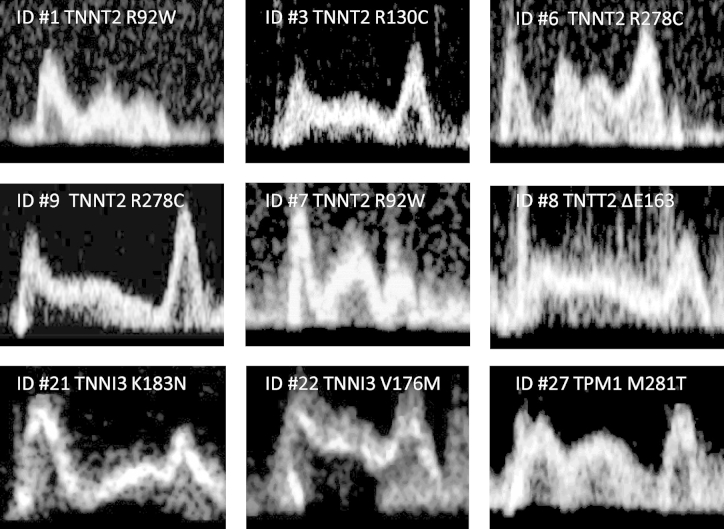
Triphasic LV Filling Pattern Representative transmitral blood flow velocity patterns assessed by pulsed-wave Doppler echocardiography in 9 patients with thin-filament–associated hypertrophic cardiomyopathy (patient ID numbers and mutations are shown). Mid-diastolic flow velocity (L-wave) is clearly visible (*). An L wave’s presence is independent of the overall diastolic pattern, with E/A ratios that can be <1 (i.e., delayed relaxation) or >1 (i.e., pseudonormalized). LV = left ventricular.

Transmitral pulsed-wave interrogation showed a triphasic LV filling pattern characterized by an L-wave with prominent mid-diastolic flow velocity in 26% of thin-filament patients, but in only 11% with thick-filament disease (p = 0.002) (Figure 4). Furthermore, early diastolic lateral mitral annulus velocity (E′) was 24% lower in patients with thin compared with thick-filament mutations (p < 0.001) (Table 1).

CMR studies in 47 thin-filament patients (59%) and 76 thick-filament patients (51%) highlighted significant differences between these cohorts. Thin-filament patients had a smaller LV mass index and a lower LV ejection fraction (Table 1). Although LGE was present in the majority of patients from both cohorts, the proportion of LV mass occupied was larger in thin-filament patients (20 ± 11% vs. 16 ± 8% in thick-filament patients). LGE exceeded 30% of LV mass in 12 thin-filament patients (27%), 10 of whom showed an EF <50% or a restrictive diastolic pattern at final evaluation. Only 8 thick-filament patients (11%) showed LGE exceeding 30% of the LV.

#### SCD risk profile

Compared with the thick-filament cohort, thin-filament patients had a higher prevalence of NSVT, abnormal blood pressure response to exercise, and family history of SCD and were more likely to have at least 1 established SCD risk factor (74% vs. 59%; p = 0.031) (Table 1). However, the proportion of patients with 2 or more risk factors was similar in both groups (30% vs. 34%, respectively, p = 0.39).

### Clinical outcomes and symptomatic progression

Mean follow-up for the thin-filament cohort was 4.7 ± 2.7 years, for a total of 361 patient-years, comparable to the thick-filament group (4.7 ± 3.0 years, p = 0.49). During this time, 2 patients from the thin-filament cohort (2.5%) died of cardiac causes (1 suddenly, 1 because of heart failure), 3 (4%) experienced nonfatal strokes, 3 (4%) had resuscitated cardiac arrests, and 5 (6%) had appropriate implantable cardioverter-defibrillator (ICD) shocks owing to rapid ventricular tachycardia or fibrillation (Table 2). All-cause mortality, cardiac mortality, and SCD rates did not differ between the cohorts (p > 0.05 for all comparisons) (Table 2), and their rates of malignant arrhythmias (including sudden death, resuscitated cardiac arrest, and appropriate ICD shocks) were also similar (Table 2).

**Table 2:** Management and Clinical Outcomes

	Thin Filament (n = 80)	Thick Filament (n = 150)	p Value
Follow-up, yrs	4.7 ± 2.7	4.7 ± 3.0	0.492
Clinical outcomes			
HCM-related death	2 (2)	10 (6)	0.167
Heart failure related	1 (1)	1 (1)	0.321
Sudden-unexpected	1 (1)	9 (7)	0.091
Resuscitated cardiac arrest	4 (5)	3 (2)	0.241
Appropriate ICD shocks	6 (8)	5 (3)	0.158
Total with malignant arrhythmias∗	11 (14)	17 (11)	0.593
Nonfatal stroke	3 (4)	6 (4)	0.542
NYHA functional class at final evaluation			
I	41 (51)	79 (53)	0.267
II	24 (30)	56 (37)	0.750
III/IV	16 (20)	15 (10)	0.034
Progression to NYHA functional class III or IV	12 (15)	8 (5)	0.013
New-onset AF	9 (11)	14 (9)	0.527
Final echocardiographic evaluation			
LVEF, %	60 ± 10	63 ± 11	0.043
With LVEF <50%	14 (18)	12 (8)	0.031
LV filling pattern			
Normal	17 (21)	32 (26)	0.406
Impaired relaxation	19 (24)	45 (36)	0.113
Pseudonormalized	27 (34)	41 (33)	0.362
Restrictive	13 (16)	7 (5)	0.003
With progression to EF <50%/restrictive diastole	16 (20)	14 (9)	0.038
Moderate/severe left atrial dilation†	40 (50)	51 (34)	0.023
Interventions			
Implantable cardioverter-defibrillator	19 (24)	36 (24)	0.516
Catheter ablation for AF	10 (12)	8 (5)	0.040
Alcohol ablation or myectomy	11 (14)	38 (25)	0.041
Pharmacological therapy			
On treatment	75 (94)	141 (94)	0.503
Beta-blockers	54 (67)	113 (75)	0.411
Verapamil	20 (25)	8 (5)	<0.001
Amiodarone	14 (18)	29 (19)	0.383
Disopyramide	2 (3)	34 (23)	<0.001
Diuretics	22 (27)	23 (15)	0.038
ACE inhibitors or ARB	27 (34)	31 (21)	0.033
Warfarin	17 (21)	21 (14)	0.102

Values are mean ± SD or n (%).

ACE = angiotensin-converting enzyme; AF = atrial fibrillation; ARB = angiotensin receptor blockers; EF = ejection fraction; LVEF = left ventricular ejection fraction; ICD = implantable cardioverter-defibrillator; other abbreviations as in Table 1.

∗ Including sudden cardiac death, resuscitated cardiac arrest, and appropriate ICD shocks.

† Left atrial diameter >45 mm in men or >42 mm in women.

**Figure 5:**
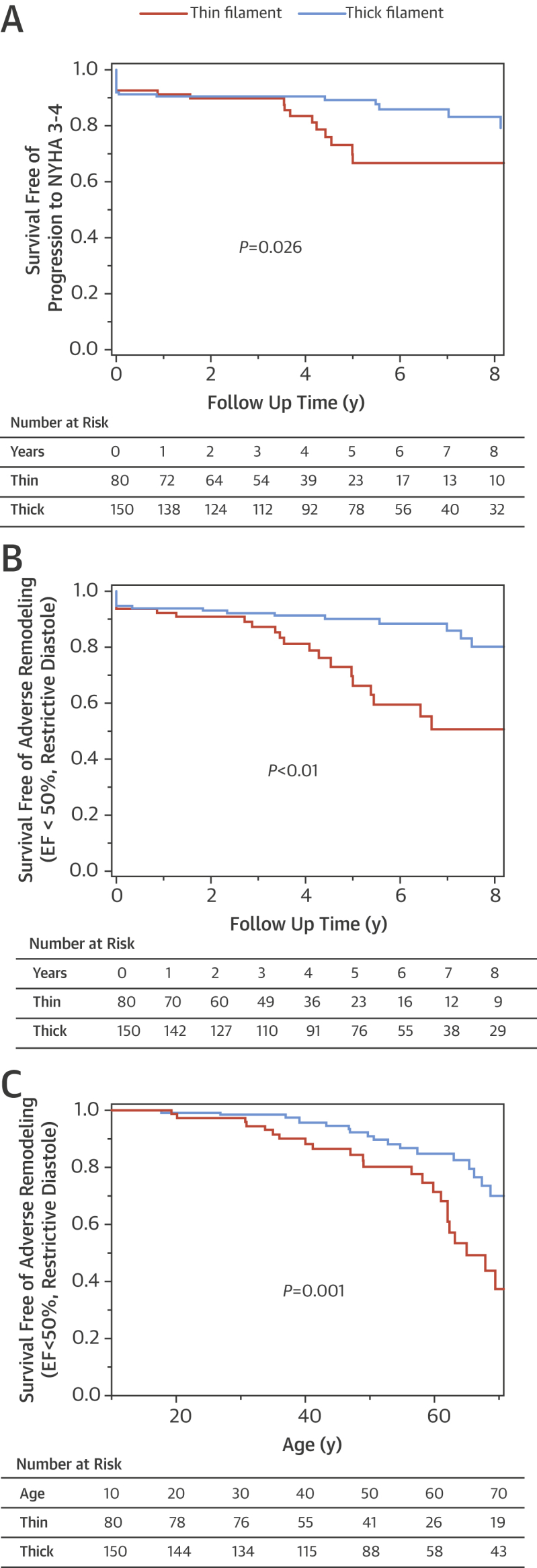
Clinical and Instrumental Outcomes in Thick- Versus Thin-Filament HCM **(A)** Kaplan-Mayer curve illustrating survival free of progression to severe heart failure (New York Health Association [NYHA] functional classes III/IV). **(B)** Survival free of adverse left ventricular (LV) remodeling and dysfunction during follow-up, defined as progression to LV ejection fraction (EF) <50% or toward restrictive LV filling pattern. **(C)** Lifelong likelihood of advanced LV dysfunction (defined as in **B**) in relation to genetic status. The probability values are calculated with the log-rank test comparing thin-filament versus thick-filament survival curves. HCM = hypertrophic cardiomyopathy; NYHA = New York Heart Association.

At final evaluation, moderate or severe congestive symptoms (NYHA functional class III/IV) were more prevalent in the thin compared with the thick-filament subgroup (19% vs. 10%; p = 0.034) (Table 2). Notably, 12 (15%) thin-filament patients with mild or no symptoms at initial evaluation progressed to NYHA functional class III/IV during follow-up at a mean age of 50 ± 9 years, 3 times the prevalence in the thick-filament cohort (5%; p = 0.013). Survival analysis showed a higher likelihood of developing moderate or severe congestive symptoms among thin-filament patients (Figure 5A). At multivariate analysis, performed on the 2 HCM cohorts combined, the presence of thin-filament disease more than doubled the likelihood of a final NYHA functional class III/IV (hazard ratio [HR]: 2.16, p = 0.040), independent of LV outflow obstruction (HR: 4.06; p <0.001) and AF (HR 2.74, p = 0.008) (Online Figure 1).

### Evidence of adverse LV remodeling and dysfunction

**Figure 6:**
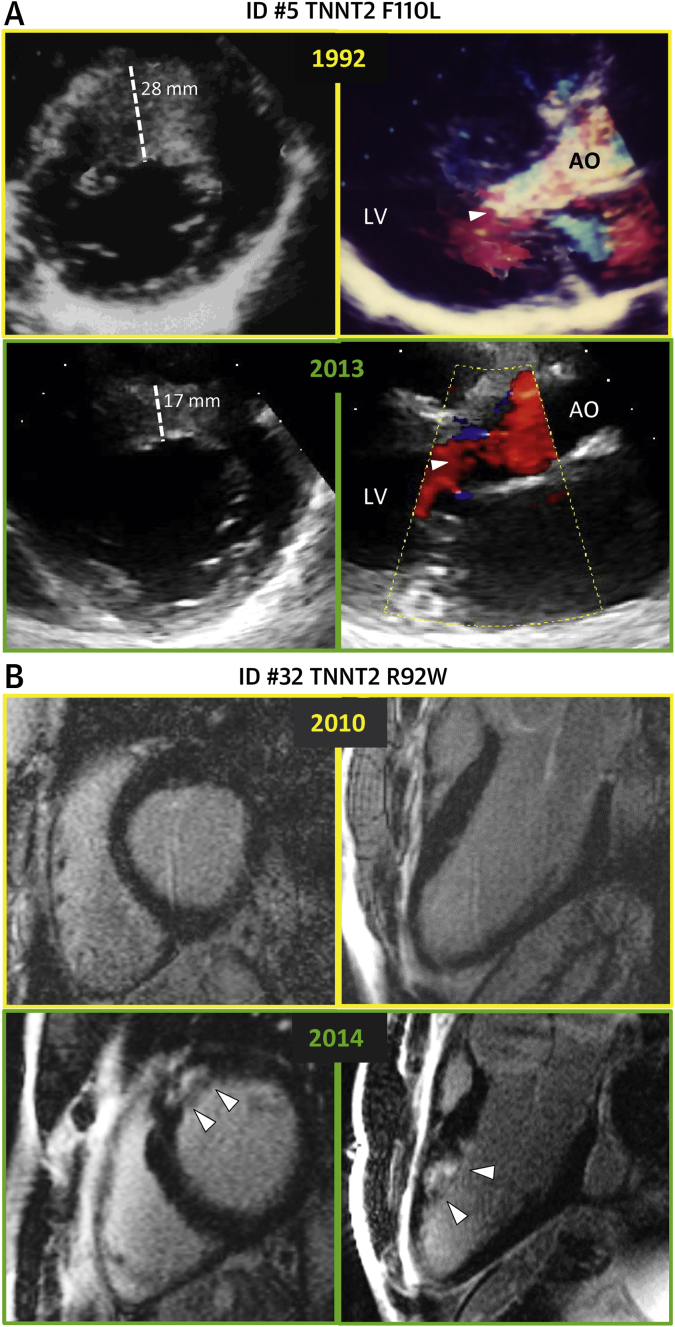
Evidence of Disease Progression in Thin-filament HCM **(A)** Echocardiographic images from patient ID #5 carrying the TNNT2-F110L mutation. **Top**: Echocardiographic evaluation at age 16 years. **(Left)** Parasternal short-axis view showing severe and diffuse anteroseptal LV hypertrophy. **(Right)** Parasternal long-axis view showing turbulent flow in the LV outflow tract **(arrow)**, caused by severe dynamic obstruction. **(Bottom)** Similar views from the same patient at age 37 years, showing marked anteroseptal wall thinning and absence of obstruction and increased left atrial size. **(B)** CMR images from patient ID #32 carrying the TNNT2-R92W mutation. **(Top)** Cardiac magnetic resonance at age 21 years. Short-axis and 3-chamber views show absence of late gadolinium enhancement (LGE) in the LV wall. **(Bottom)** Similar views from the same patient at age 25 years. LGE shows extensive fibrous substitution within the anterior septum **(arrow)**, occupying 25% of total LV mass. Abbreviations as in Figure 5.

Advanced LV dysfunction (defined as LVEF <50% or restrictive diastolic pattern) was present at final evaluation in 23 of the 80 thin-filament patients (29%) at ages ranging from 20 to 76 years, compared with 17 of the 150 thick-filament patients (p = 0.002). Of note, 10 of these 23 patients (43%) were younger than 50 years of age (Figure 6). The incidence of new LV dysfunction during follow-up proved higher in the thin-filament cohort (4.3% per annum) than in the thick-filament cohort (1.9% per annum; p = 0.013) (Figures 5B and 5C). In multivariate analysis, thin-filament mutations predicted LV dysfunction (HR: 2.28, p = 0.016) independent of female sex (HR: 2.08, p = 0.031), AF (HR: 2.33, p = 0.023), and NYHA functional class III symptoms at baseline (HR: 2.64, p = 0.044) (Online Figures 2 and 3). Consistent with the pronounced diastolic impairment, thin-filament patients more often had moderate or severe atrial dilation at final evaluation than thick-filament patients. Conversely, the incidence of new AF during follow-up was comparable in both groups (Table 2).

### Management

During follow-up, most thin-filament patients (n = 75; 94%) received pharmacological treatment for HCM, including beta-blockers, verapamil, amiodarone, and disopyramide (Table 2). Predictably, they were more frequently treated with diuretics and angiotensin-converting enzyme inhibitors or angiotensin receptor blockers than the thick-filament cohort but less often received disopyramide (Table 2).

Nineteen thin-filament patients (24%) received an ICD (Table 2), including 16 for primary and 3 for secondary prevention of SCD. Furthermore, 11 patients (14%) were referred for surgical septal myectomy (n = 7) or alcohol septal ablation (n = 4) for drug-refractory symptoms associated with LV outflow tract obstruction. Finally, 11 patients (14%) underwent radiofrequency catheter ablation for symptomatic, drug-refractory AF. Compared with thick-filament patients (Table 2), thin-filament patients more often underwent catheter ablation procedures for AF but were less frequently referred for invasive septal reduction therapies; ICD implantation rates were comparable (Table 2).

## Discussion

This study supports the hypothesis that thin-filament HCM is phenotypically distinct from the more common thick-filament HCM 4, 6, 9, 12. Specific LV morphological, functional, and remodeling differences were identified, suggesting unique underlying pathophysiological mechanisms 4, 6. At initial evaluation, thin-filament patients showed lesser LV hypertrophy, often developing in apical or concentric patterns, whereas patients with thick-filament disease almost universally displayed classic asymmetric LV hypertrophy involving the basal septum and anterior wall (25). As a result, dynamic LV outflow tract obstruction was less common in the thin-filament cohort (19%, compared with 34% among thick-filament patients, p = 0.015) (15), likely as a result of relative preservation of LV outflow morphology and function and reduced propensity to systolic anterior motion (14). Conversely, diastolic abnormalities were more common and pronounced in thin-filament HCM, including 16% of patients with restrictive LV pathophysiology. More than one-quarter of our thin-filament patients exhibited a triphasic LV filling pattern, compared with only 11% in the thick-filament subgroup, a disproportionate prevalence, consistent with severe diastolic impairment 21, 26. The presence of an L wave (a velocity peak ≥0.2 m/s during diastasis) (21), believed to indicate elevated filling pressures (27) and previously observed in HCM (28), has been associated with extensive septal fibrosis (26). Indeed, substantial LV remodeling occurred in thin-filament patients, mediated by progressive myocardial fibrosis (Figure 6). LGE was present in 85% of patients undergoing CMR and averaged 20% of the whole LV, reflecting greater prevalence and extension of fibrous tissue compared with our thick-filament patients and previously published, unselected HCM cohorts, largely reflecting thick-filament disease 29, 30.

### LV dysfunction and heart failure in thin-filament HCM

At the end of 4.7 years of follow-up, 29% of the 80 thin-filament patients had advanced LV dysfunction (defined as LVEF <50% or restrictive diastolic pattern), more than double the prevalence among thick-filament patients. Indeed, the incidence of newly occurring systolic dysfunction in our thin-filament patients was approximately 2.5% per year, compared with approximately 1% per year in our thick-filament subset; the latter value closely agrees with values in unselected HCM populations 31, 32. Furthermore, restrictive LV pathophysiology with preserved systolic function was observed in 11% of thin-filament patients during follow-up, consistent with prior reports emphasizing isolated, severe diastolic dysfunction in patients with troponin mutations, particularly troponin I, which occasionally presents as primary restrictive disease (33).

At final evaluation, the adverse remodeling process observed in thin-filament HCM patients was paralleled by a considerable prevalence of moderate or severe congestive symptoms. Overall, 15% of thin-filament patients initially presenting with mild or no symptoms progressed to NYHA functional class III/IV during follow-up, 3 times the prevalence in the thick-filament cohort (5%; p = 0.013). In a multivariate model assessing established predictors of HCM outcome, thin-filament disease more than doubled the likelihood of a final NYHA functional class III/IV, independent of LV outflow obstruction and AF. Conversely, thick- and thin-filament HCM patients had comparably low rates of malignant ventricular arrhythmias, including SCD, resuscitated cardiac arrest, and appropriate ICD interventions. This limited arrhythmic propensity contrasts with prior reports suggesting increased risk of SCD in patients with troponin T or I mutations, likely because of the malignant profile of the highly selected families in early studies (8). Although individual arrhythmic risk may vary considerably, particularly in children and adolescents, thin-filament HCM in our cohort emerged as a progressive condition characterized by adverse LV remodeling and dysfunction, rather than by enhanced arrhythmogenicity. Clinical implications include the need for heightened attention to early signs of LV dysfunction and symptom progression in patients with thin-filament HCM. Conversely, aggressive strategies for primary prevention of SCD, including ICDs, are not warranted solely because a thin-filament mutation is present 13, 23.

### Molecular consequences of thin-filament mutations

Preclinical studies using animal models support key features identified in our cohort of patients with thin-filament HCM (4). Transgenic mouse lines with thin-filament gene defects develop restrictive diastolic patterns and systolic dysfunction over time (34). Skinned myocardial tissue from patient samples and animal models with thin-filament mutations consistently show markedly increased myofilament Ca^2+^ sensitivity (35), closely related to abnormalities of cardiac relaxation (36) and diastolic dysfunction. Furthermore, thin-filament defects can alter cardiac function by increasing the energy cost of contraction (37). Although several of these abnormalities are shared with defects in other HCM-related genes, their extent is generally greater in thin-filament HCM samples. The constellation of early impairment in excitation-contraction coupling, energetic derangement, abnormal cardiomyocyte signaling, and intrinsic abnormalities of sarcomeric relaxation caused by thin-filament mutations may collectively drive aggressive remodeling at the cellular and extracellular levels (6), resulting in impaired contractile and relaxation properties of the myocardium 6, 38; these ultimately account for the common occurrence of progressive LV dysfunction observed in our HCM cohort.

## Conclusions

Distinctive clinical and biophysical features characterize HCM associated with thin-filament mutations, at variance with the more common thick-filament disease. Thin-filament HCM is associated with less prominent and atypically distributed LV hypertrophy, increased LV fibrosis, higher likelihood of adverse LV remodeling leading to functional deterioration, and more frequent occurrence of triphasic LV filling, reflecting profound diastolic dysfunction (Central Illustration). Management strategies should consider adequate surveillance for early detection of LV dysfunction and symptomatic progression. Conversely, arrhythmic risk does not appear to increase solely as a result of a thin-filament genotype.

**Central Illustration:**
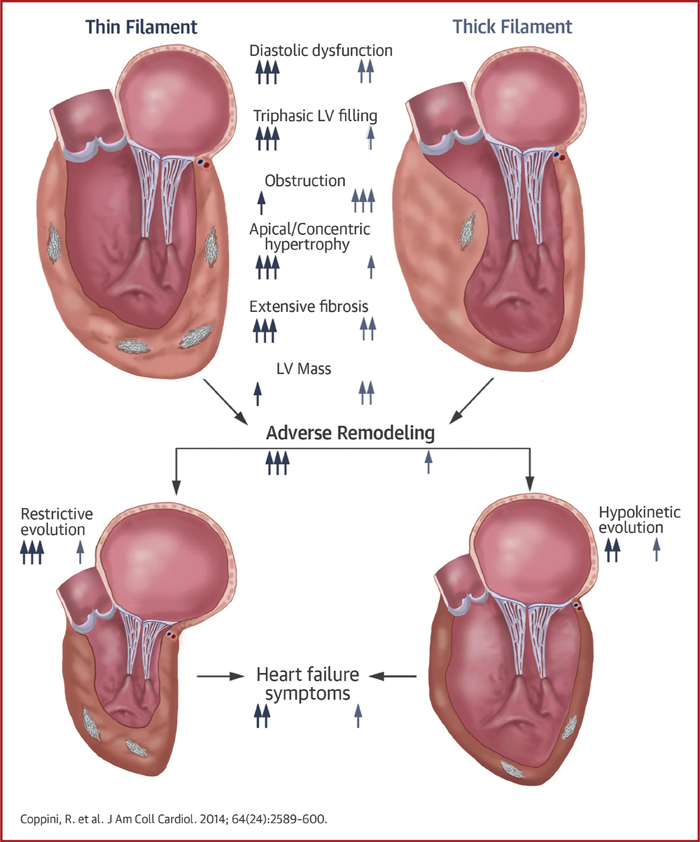
Morphological and Functional Features and Outcomes of HCM Owing to Thin-Filament Mutations **(Top)** Hearts with thin-filament **(left, blue/gray)** and thick-filament **(right, light blue/gray)** mutations, highlighting the main morphological and functional features of the 2 groups (**1 arrow** = a poorly represented feature, **2 arrows** = a moderately represented feature, and **3 arrows** = a highly prevalent feature). **(Bottom)** Outcome of thin-filament and thick-filament HCM. Adverse remodeling is more common in thin-filament patients and may lead to either restrictive or hypokinetic morphological and functional end-stage phenotypes. Both are more represented in thin-filament patients and are related to increased prevalence of heart failure symptoms in the thin-filament cohort. HCM = hypertrophic cardiomyopathy; LV = left ventricular.

Perspectives **COMPETENCY IN MEDICAL KNOWLEDGE:** HCM associated with thin-filament mutations is characterized by less prominent and atypically distributed hypertrophy; increased fibrosis; and more adverse remodeling (hypokinetic or restrictive evolution), leading to congestive symptoms, triphasic left ventricular filling, and more severe diastolic dysfunction, compared with thick-filament HCM.

**COMPETENCY IN PATIENT CARE:** Aggressive measures for primary prevention of sudden death with implanted cardiac defibrillators in patients with HCM should not be based solely on genotype.

**TRANSLATIONAL OUTLOOK 1:** Better understanding of early phenotype development in thin-filament cardiomyopathy will require studies of index patients’ families, including monitoring of young relatives carrying thin-filament mutations over a relatively long period and comparison with carriers of thick-filament mutations.

**TRANSLATIONAL OUTLOOK 2:** Preclinical studies involving transgenic animal models are warranted to assess mechanisms of disease progression and to test pharmacological strategies for controlling symptoms and reducing adverse myocardial remodeling in thin-filament HCM.
