# Supplementary material for: Clinical Phenotype and Outcome of Hypertrophic Cardiomyopathy Associated With Thin-Filament Gene Mutations
Source: J Am Coll Cardiol. 2014 Dec 23;64(24):2589–600. doi: 10.1016/j.jacc.2014.09.059 (PMC4270453; doi:10.1016/j.jacc.2014.09.059)
Supplement: Online Tables 1–4 and Online Figures 1–3 [file mmc1.pdf]

Supplementary Table 1: mutational analysis of thin filament patients

Pt. ID	Gene	C	gNomen	Transcript	cNomen	Exon/ Intron	pNomen	Novelty	db SNP	Classification	Fam. Segregation	N conservation	AA conservation	AA wt	AA mut	Grantham score	AGVGD class	SIFT (score)	POLYPHEN (score)	Other Publications
1	TNNT2	1	g.199601049C>T	NM_001001430.1	c.274C>T	9	p.Arg92Trp	Moolman (1997) J Am Coll Cardiol	no	pathogenic	-	Highly (1.0)	Highly	basic	hydrophobic	101	C0	affect protein function (0.00)	probably damaging (1.000)	Revera(2008) Cardiovasc R Pasquale (2011) Circ cardiovasc Gen
2	TNNT2	1	g.199601048G>A	NM_001001430.1	c.275G>A	9	p.Arg92Gln	Thierfelder (1994) Cell	no	pathogenic	-	Highly (1.0)	Highly	basic	polar uncharged	43	C0	affect protein function (0.00)	probably damaging (1.000)	Yanaga (1999) J Biol Chem Watkins H (1995) NEJM Torricelli F, Am J Cardiol 2003
3	TNNT2	1	g.199600120C>T	NM_001001430.1	c.388C>T	11	p.Arg130Cys	Torricelli F, Am J Cardiol 2003	no	pathogenic	-	Highly (0.9)	Highly	basic	hydrophobic	180	C65	affect protein function (0.00)	probably damaging (1.000)	Song L (2005) Clin Chim Acta
4	TNNT2	1	g.199600120C>T	NM_001001430.1	c.388C>T	11	p.Arg130Cys	Torricelli F, Am J Cardiol 2003	no	pathogenic	-	Highly (0.9)	Highly	basic	hydrophobic	180	C65	affect protein function (0.00)	probably damaging (1.000)	Song L (2005) Clin Chim Acta
5	TNNT2	1	g.199600993T>G	NM_001001430.1	c.330T>G	10	p.Phe110Leu	Torricelli F, Am J Cardiol 2003	no	pathogenic	yes	Highly (1.0)	Highly	hydrophobic	hydrophobic	22	C0	tolerated (0.77)	probably damaging (1.000)	Tao Q (2007) Journal of Clinical Rehabilitative Tissue Engineering Research
6	TNNT2	1	g.199594996C>T	NM_001001430.1	c.832C>T	17	p.Arg278Cys	Watkins (1995) N Engl J Med	yes	pathogenic	yes	Highly (1.0)	Weakly	basic	hydrophobic	180	C0	affect protein function (0.00)	probably damaging (1.000)	Yanaga (1999) J Biol Chem Pasquale (2011) Circ cardiovasc Gen
7	TNNT2	1	g.199601049C>T	NM_001001430.1	c.274C>T	10	p.Arg92Trp	Moolman (1997) J Am Coll Cardiol	no	pathogenic	-	Highly (1.0)	Highly	basic	hydrophobic	101	C0	affect protein function (0.00)	probably damaging (1.000)	Revera(2008) Cardiovasc R Pasquale (2011) Circ CV Gen
8	TNNT2	1	g.199599128_130del	NM_001001430.1	c.487_489del	12	p.Glu163del	Richard P (2003) Circulation	no	pathogenic	-	-	-	-	-	-	-	-	-	Millat (2010) Eur J Med Genet.
9	TNNT2	1	g.199594996C>T	NM_001001430.1	c.832C>T	17	p.Arg278Cys	Watkins (1995) N Engl J Med	yes	pathogenic	yes	Highly (1.0)	Weakly	basic	hydrophobic	180	C0	affect protein function (0.00)	probably damaging (1.000)	Yanaga (1999) J Biol Chem Pasquale (2011) Circ CV Gen
10	TNNT2	1	g.199600993T>G	NM_001001430.1	c.330T>G	10	p.Phe110Leu	Torricelli F, Am J Cardiol 2003	no	pathogenic	yes	Highly (1.0)	Highly	hydrophobic	hydrophobic	22	C0	tolerated (0.77)	probably damaging (1.000)	Tao Q (2007) Journal of Clinical Rehabilitative Tissue Engineering Research
11	TNNT2	1	g.199600993T>G	NM_001001430.1	c.330T>G	10	p.Phe110Leu	Torricelli F, Am J Cardiol 2003	no	pathogenic	yes	Highly (1.0)	Highly	hydrophobic	hydrophobic	22	C0	tolerated (0.77)	probably damaging (1.000)	Tao Q (2007) Journal of Clinical Rehabilitative Tissue Engineering Research
12	TNNT2	1	g.199600120C>T	NM_001001430.1	c.388C>T	11	p.Arg130Cys	Torricelli F, Am J Cardiol 2003	no	pathogenic	-	Highly (0.9)	Highly	basic	hydrophobic	180	C65	affect protein function (0.00)	probably damaging (1.000)	Song L (2005) Clin Chim Acta
13	TNNT2	1	g.199600120C>T	NM_001001430.1	c.388C>T	11	p.Arg130Cys	Torricelli F, Am J Cardiol 2003	no	pathogenic	-	Highly (0.9)	Highly	basic	hydrophobic	180	C65	affect protein function (0.00)	probably damaging (1.000)	Song L (2005) Clin Chim Acta
14	TNNT2	1	g.199601049C>T	NM_001001430.1	c.274C>T	9	p.Arg92Trp	Moolman (1997) J Am Coll Cardiol	no	pathogenic	-	Highly (1.0)	Highly	basic	hydrophobic	101	C0	affect protein function (0.00)	probably damaging (1.000)	Revera(2008) Cardiovasc R Pasquale (2011) Circ CV Gen
15	TNNT2	1	g.199594972G>A	NM_001001430.1	c.856C>T	17	p.Arg286Cys	Richard P (2003)Circulation	no	pathogenic	yes	Highly (1.0)	Moderately	basic	polar uncharged	180	C0	affect protein function (0.00)	probably damaging (1.000)	Miliou (2005) Heart
16	TNNT2	1	g.199601048G>A	NM_001001430.1	c.275G>A	9	p.Arg92Gln	Thierfelder (1994) Cell	no	pathogenic	-	Highly (1.0)	Highly	basic	polar uncharged	43	C0	affect protein function (0.00)	probably damaging (1.000)	Yanaga (1999) J Biol Chem Watkins H (1995) NEJM Torricelli F, Am J Cardiol 2003
17	TNNT2	1	g.199594972G>A	NM_001001430.1	c.856C>T	17	p.Arg286Cys	Richard P (2003)Circulation	no	pathogenic	yes	Highly (1.0)	Moderately	basic	polar uncharged	180	C0	affect protein function (0.00)	probably damaging (1.000)	Miliou (2005) Heart
18	TNNI3	19	g.55663278C>T	NM_000363.4	c.557G>A	8	p.Arg186Gln	Richard P (2003)Circulation	no	pathogenic	yes	Weakly (0.0)	Highly	basic	polar uncharged	43	C0	tolerated (0.07)	possibly damaging (0.899)	Mogensen J (2004)J Am Coll Cardiol.
19	TNNI3	19	g.60355066A>G	NM_000363.4	c.581A>G	8	p.Asn194Ser	yes	no	likely pathogenic	?	Highly (1.0)	Highly	polar uncharged	polar uncharged	46	C0	affect protein function (0.00)	probably damaging (0.993)	
20	TNNI3	19	g.60357210G>T	NM_000363.4	c.549G>T	7	p.Lys183Asn	yes	no	likely pathogenic	?	Highly (1.0)	Moderately	basic	polar uncharged	94	C0	affect protein function (0.00)	possibly damaging (0.902)	
21	TNNI3	19	g.60357210G>T	NM_000363.4	c.549G>T	7	p.Lys183Asn	yes	no	likely pathogenic	?	Highly (1.0)	Moderately	basic	polar uncharged	94	C0	affect protein function (0.00)	possibly damaging (0.902)	

Pt. ID	Gene	C	gNomen	Transcript	cNomen	Exon/ Intron	pNomen	Novelty	db SNP	Classification	Fam. Segregation	N conservation	AA conservation	AA wt	AA mut	Grantham score	AGVGD class	SIFT (score)	POLYPHEN (score)	Other Publications
22	TNNI3	19	g.60357233G>A	NM_000363.4	c.526G>A	7	p.Val176Met	yes	no	likely pathogenic	?	Highly (1.0)	Highly	hydrophobic	hydrophobic	21	C0	affect protein function (0.00)	probably damaging (0.964)	
23	TNNI3	19	g.60355066A>G	NM_000363.4	c.592C>G	8	p.Leu198Val	Merk, Seidman et al. (2005) Cardiogenomics	no	pathogenic	-	Weakly (0.0)	Highly	hydrophobic	hydrophobic	32	C0	affect protein function (0.00)	possibly damaging (0.767)	Morita (2002). Cold Spring Harb Symp Quant Biol.
24	TNNI3	19	g.60355066A>G	NM_000363.4	c.620A>C	8	p.Lys207Thr	yes	no	likely pathogenic	yes	Highly (1.0)	Highly	basic	polar uncharged	78	C0	affect protein function (0.00)	possibly damaging (0.895)	
25	TNNI3	19	g.60355066A>G	NM_000363.4	c.592C>G	8	p.Leu198Val	Merk, Seidman et al. (2005) Cardiogenomics	no	pathogenic	-	Weakly (0.0)	Highly	hydrophobic	hydrophobic	32	C0	affect protein function (0.00)	possibly damaging (0.767)	Morita (2002). Cold Spring Harb Symp Quant Biol.
26	TPM1	15	g.61143385T>C	NM_001018005.1	c.842T>C	9	p.Met281Thr	yes	no	likely pathogenic	yes	Highly (1.0)	Weakly	hydrophobic	polar uncharged	81	C45	affect protein function (0.01)	benign (0.024)	
27	TPM1	15	g.61143385T>C	NM_001018005.1	c.842T>C	9	p.Met281Thr	yes	no	likely pathogenic	yes	Highly (1.0)	Weakly	hydrophobic	polar uncharged	81	C45	affect protein function (0.01)	benign (0.024)	
28	ACTC1	15	g.32874235T>C	NM_005159.4	c.67T>C	2	p.Phe23Leu	yes	no	likely pathogenic	yes	Highly (1.0)	Highly	hydrophobic	hydrophobic	22	C15	affect protein function (0.00)	benign (0.231)	
29	ACTC1	15	g.32874235T>C	NM_005159.4	c.67T>C	2	p.Phe23Leu	yes	no	likely pathogenic	yes	Highly (1.0)	Highly	hydrophobic	hydrophobic	22	C15	affect protein function (0.00)	benign (0.231)	
30	ACTC1	15	g.32870779C>T	NM_005159.4	c.818C>T	6	p.Ser273Phe	yes	no	likely pathogenic	?	Highly (1.0)	Highly	polar unchraged	hydrophobic	155	C25	affect protein function (0.00)	possibly damaging (0.933)	
31	TNNT2	1	g.199601049C>T	NM_001001430.1	c.274C>T	9	p.Arg92Trp	Moolman (1997) J Am Coll Cardiol	no	pathogenic	-	Highly (1.0)	Highly	basic	hydrophobic	101	C0	affect protein function (0.00)	probably damaging (1.000)	Revera(2008) Cardiovasc R Pasquale (2011) Circ CV Gen
32	TNNT2	1	g.199601049C>T	NM_001001430.1	c.274C>T	9	p.Arg92Trp	Moolman (1997) J Am Coll Cardiol	no	pathogenic	-	Highly (1.0)	Highly	basic	hydrophobic	101	C0	affect protein function (0.00)	probably damaging (1.000)	Revera(2008) Cardiovasc R Pasquale (2011) Circ CV Gen
33	TPM1	15	g.63335086G>A	NM_001018005.1	c.58G>A	1	p.Asp20Asn	yes	no	likely pathogenic	-	Highly (1.0)	Highly	acidic	polar uncharged	23	C15	affect protein function (0.00)	benign (0.019)	
34	TPM1	15	g.63354429G>A	NM_001018005.1	c.655G>A	7	p.Asp219Asn	yes	no	likely pathogenic	-	Highly (1.0)	Highly	acidic	polar uncharged	23	C15	affect protein function (0.00)	benign (0.104)	
35	TNNT2	1	g.199595410T>C	NM_001001430.1	c.785A>G	16	p.Asn262Ser	yes	no	likely pathogenic		Highly (1.0)	Moderately	polar uncharged	polar uncharged	46	C0	tolerated (0.21)	possibly damaging	
36	TNNT2	1	g.199595410T>C	NM_001001430.1	c.785A>G	16	p.Asn262Ser	yes	no	likely pathogenic		Highly (1.0)	Moderately	polar uncharged	polar uncharged	46	C0	tolerated (0.21)	possibly damaging	
37	TNNI3	19	g.55665462C>G	NM_000363.4	c.485G>C	7	p.Arg162Pro	Richard P (2003) Circulation	no	pathogenic	yes	Moderately	Moderately	basic	hydrophobic	103	C0	affect protein function (0.00)	possibly damaging (1.804)	Doolan (2005) JMCC Ingles J (2005) J Med Genet
38	TNNT2	1	g.199594996C>T	NM_001001430.1	c.832C>T	17	p.Arg278Cys	Watkins (1995) N Engl J Med	yes	pathogenic	yes	Highly (1.0)	Weakly	basic	hydrophobic	180	C0	affect protein function (0.00)	probably damaging (1.000)	Yanaga (1999) J Biol Chem Pasquale (2011) Circ CV Gen
39	TNNT2	1	g.199594972G>A	NM_001001430.1	c.856C>T	17	p.Arg286Cys	Richard P (2003)Circulation	no	pathogenic	yes	Highly (1.0)	Moderately	basic	polar uncharged	180	C0	affect protein function (0.00)	probably damaging (1.000)	Miliou (2005) Heart
40	TNNT2	1	g.199594996C>T	NM_001001430.1	c.832C>T	17	p.Arg278Cys	Watkins (1995) N Engl J Med	yes	pathogenic	yes	Highly (1.0)	Weakly	basic	hydrophobic	180	C0	affect protein function (0.00)	probably damaging (1.000)	Yanaga (1999) J Biol Chem Pasquale (2011) Circ CV Gen
41	TNNT2	1	g.199594996C>T	NM_001001430.1	c.832C>T	17	p.Arg278Cys	Watkins (1995) N Engl J Med	yes	pathogenic	yes	Highly (1.0)	Weakly	basic	hydrophobic	180	C0	affect protein function (0.00)	probably damaging (1.000)	Yanaga (1999) J Biol Chem Pasquale (2011) Circ CV Gen
42	TNNT2	1	g.199601042C>T	NM_001001430.1	c.281G>A	10	p.Arg94His	yes	no	likely pathogenic	?	Highly (1.0)	Highly	basic	basic	29	C0	tolerated (0.33)	probably damaging (1.000)	

Pt. ID	Gene	C	gNomen	Transcript	cNomen	Exon/ Intron	pNomen	Novelty	db SNP	Classification	Fam. Segregation	N conservation	AA conservation	AA wt	AA mut	Grantham score	AGVGD class	SIFT (score)	POLYPHEN (score)	Other Publications
43	TNNT2	1	g.199595388G>C	NM_001001430.1	c.807C>G	16	p.Asn269Lys	Heba(2013) J. Cardiovasc.Trans. Res	no	likely pathogenic	?	Highly (1.0)	Weakly	polar uncharged	basic	94	C0	tolerated (1.00)	benign (0.048)	
44	TNNI3	19	g.55665514G>A	NM_000363.4	c.433C>T	7	p.Arg145Trp	Mogensen J (2004) J Am Coll Cardiol	yes	pathogenic	yes	Weakly (0.0)	Moderately	basic	hydrophobic	101	C0	affect protein function (0.00)	probably damaging (1.000)	Cheng (2005) J Am Coll Card van den Wijngaard (2011) Neth Heart J
45	TNNI3	19	g.55665514G>A	NM_000363.4	c.433C>T	7	p.Arg145Trp	Mogensen J (2004) J Am Coll Cardiol	yes	pathogenic	yes	Weakly (0.0)	Moderately	basic	hydrophobic	101	C0	affect protein function (0.00)	probably damaging (1.000)	Cheng (2005) J Am Coll Card van den Wijngaard (2011) Neth Heart J
46	TNNI3	19	g.55665462C>T	NM_000363.4	c.485G>A	7	p.Arg162Gln	Van Driest SL (2003) Circulation	no	pathogenic	-	Moderately	Moderately	basic	polar uncharged	43	C0	tolerated (0.11)	possibly damaging (0.768)	Mogensen(2004) JACC Cheng (2005) JACC
47	TPM1	15	g.63353922G>A	NM_001018005.1	c.574G>A	9	p.Glu192Lys	Probst S (2011) Circ CardiovascGenet	yes	pathogenic	-	Highly (1.0)	Highly	acidic	basic	56	C55	affect protein function (0.00)	benign (0.013)	Probst (2001) Circ Cardiovasc Gen
48	TNNT2	1	g.199601389A>T	NM_001001430.1	c.236T>A	9	p.Ile79Asn	Watkins (1995) N Engl J Med	no	pathogenic	yes	Highly (1.0)	Highly	hydrophobic	polar uncharged	149	C0	tolerated (0.12)	probably damaging (1.000)	Palm (2001) Biophys J Varnava (2001) Circulation Pasquale (2011) Circ CV Gen
49	TNNT2	1	g.199601389A>T	NM_001001430.1	c.236T>A	9	p.Ile79Asn	Watkins (1995) N Engl J Med	no	pathogenic	yes	Highly (1.0)	Highly	hydrophobic	polar uncharged	149	C0	tolerated (0.12)	probably damaging (1.000)	Palm (2001) Biophys J Varnava (2001) Circulation Pasquale (2011) Circ CV Gen
50	TNNT2	1	g.199601048G>A	NM_001001430.1	c.275G>A	9	p.Arg92Gln	Thierfelder (1994) Cell	no	pathogenic	-	Highly (1.0)	Highly	basic	polar uncharged	43	C0	affect protein function (0.00)	probably damaging (1.000)	Yanaga (1999) J Biol Chem Watkins H (1995) NEJM Torricelli F, Am J Cardiol 2003
51	TNNI3	19	g.55665462C>T	NM_000363.4	c.485G>A	7	p.Arg162Gln	Van Driest SL (2003) Circulation	no	pathogenic	-	Moderately	Moderately	basic	polar uncharged	43	C0	tolerated (0.11)	possibly damaging (0.768)	Mogensen(2004) JACC Cheng (2005) JACC
52	TNNT2	1	g.199599128_199599130del	NM_001001430.1	c.487_489del	12	p.Glu163del	Richard P (2003) Circulation	no	pathogenic	-	-	-	-	-	-	-	-	-	Millat (2010) Eur J M Genet. Pasquale (2011) Circ CV Gen
53	TNNT2	1	g.199601368T>G	NM_001001430.1	c.257A>C	9	p.Asp86Ala	Van Driest SL (2003) Circulation	no	pathogenic	-	Highly (1.0)	Highly	acidic	hydrophobic	126	C65	affect protein function (0.00)	probably damaging (1.000)	Bos (2014) Mayo Clin Proc
54	TNNT2	1	g.199601389A>T	NM_001001430.1	c.236T>A	9	p.Ile79Asn	Watkins (1995) N Engl J Med	no	pathogenic	yes	Highly (1.0)	Highly	hydrophobic	polar uncharged	149	C0	tolerated (0.12)	probably damaging (1.000)	Palm (2001) Biophys J Varnava (2001) Circulation Pasquale (2011) Circ CV Gen
55	TNNT2	1	g.199594972G>A	NM_001001430.1	c.856C>T	17	p.Arg286Cys	Richard P (2003)Circulation	no	pathogenic	yes	Highly (1.0)	Moderately	basic	polar uncharged	180	C0	affect protein function (0.00)	probably damaging (1.000)	Miliou (2005) Heart
56	TNNT2	1	g.199594972G>A	NM_001001430.1	c.856C>T	17	p.Arg286Cys	Richard P (2003)Circulation	no	pathogenic	yes	Highly (1.0)	Moderately	basic	polar uncharged	180	C0	affect protein function (0.00)	probably damaging (1.000)	Miliou (2005) Heart
57	TNNI3	19	g.55665525C>T	NM_000363.4	c.422G>A	7	p.Arg141Gln	Richard P (2003) Circulation	no	pathogenic	-	Weakly (0.0)	Moderately	basic	polar uncharged	43	C0	tolerated (0.05)	probably damaging (0.975)	Mogensen(2004) JACC Van Driest (2003) Circulation Bos (2014) Mayo Clin Proc
58	TNNI3	19	g.55665477G>A	NM_000363.4	c.470C>T	7	p.Ala157Val	Richard P (2003) Circulation	no	pathogenic	-	Moderately	Moderately	hydrophobic	hydrophobic	64	C0	tolerated (0.20)	possibly damaging (0.860)	Mogensen(2004) JACC Bos (2014) Mayo Clin Proc
59	ACTC1	15	g.35084404G>A	NM_005159.4	c.695C>T	5	p.Ala232Val	yes	no	likely pathogenic	-	Highly (1.0)	Highly	hydrophobic	hydrophobic	64	C65	affect protein function (0.00)	possibly damaging (0.911)	
60	TNNT2	1	g.199600993T>G	NM_001001430.1	c.330T>G	10	p.Phe110Leu	Torricelli F, Am J Cardiol 2003	no	pathogenic	yes	Highly (1.0)	Highly	hydrophobic	hydrophobic	22	C0	tolerated (0.77)	probably damaging (1.000)	Tao Q (2007) Journal of Clinical Rehabilitative Tissue Engineering Research
61	TPM1	15	g.63353922G>A	NM_001018005.1	c.574G>A	9	p.Glu192Lys	Merk, Seidman et al. (2005) Cardiogenomics	yes	pathogenic	-	Highly (1.0)	Highly	acidic	basic	56	C55	affect protein function (0.00)	benign (0.013)	Probst S (2011) Circ Cardiovasc Genet
62	TNNI3	19	g.55668509A>T	NM_000363.4	c.25-8T>A	IVS3	p.?	Kimura A (1997) Nat Genetics	yes	pathogenic	-	-	-	-	-	-	-	-	-	Murakami C (2010) Leg Med (Tokyo)
63	TNNI3	19	g.55665462C>T	NM_000363.4	c.485G>A	7	p.Arg162Gln	Van Driest SL (2003) Circulation	no	pathogenic	-	Moderately	Moderately	basic	polar uncharged	43	C0	tolerated (0.11)	possibly damaging (0.768)	Mogensen(2004) JACC Doolan (2005) JMCC Ingles J (2005) J Med Genet

Pt. ID	Gene	C	gNomen	Transcript	cNomen	Exon/ Intron	pNomen	Novelty	db SNP	Classification	Fam. Segregation	N conservation	AA conservation	AA wt	AA mut	Grantham score	AGV GD class	SIFT (score)	POLYPHEN (score)	Other Publications
64	TNNI3	19	g.55668509A>T	NM_000363.4	c.25-8T>A	IVS3	p.?	Kimura A (1997) Nat Genetics	yes	pathogenic	-	-	-	-	-	-	-	-	-	Murakami C (2010) Leg Med (Tokyo)
65	TNNI3	19	g.55668509A>T	NM_000363.4	c.25-8T>A	IVS3	p.?	Kimura A (1997) Nat Genetics	yes	pathogenic	-	-	-	-	-	-	-	-	-	Murakami C (2010) Leg Med (Tokyo)
66	TNNI3	19	g.55668509A>T	NM_000363.4	c.25-8T>A	IVS3	p.?	Kimura A (1997) Nat Genetics	yes	pathogenic	-	-	-	-	-	-	-	-	-	Murakami C (2010) Leg Med (Tokyo)
67	TNNT2	1	g.199600993T>G	NM_001001430.1	c.330T>G	10	p.Phe110Leu	Torricelli F, Am J Cardiol 2003	no	pathogenic	yes	Highly (1.0)	Highly	hydrophobic	hydrophobic	22	C0	tolerated (0.77)	probably damaging (1.000)	Tao Q (2007) Journal of Clinical Rehabilitative Tissue Engineering Research
68	TNNT2	1	g.199599158C>T	NM_001001430.1	c.460-1G>A	IVS11	p.?	yes	no	likely pathogenic	-	-	-	-	-	-	-	-	-	
69	TPM1	15	g.63335092G>A	NM_001018005.1	c.64G>A	1	p.Ala22Thr	yes	no	likely pathogenic	-	Highly (1.0)	Highly	hydrophobic	polar uncharged	58	C55	affect protein function (0.00)	probably damaging (1.000)	
70	TNNI3	19	g.55665462C>G	NM_000363.4	c.485G>C	7	p.Arg162Pro	Richard P (2003) Circulation	no	pathogenic	yes	Moderately	Moderately	basic	hydrophobic	103	C0	affect protein function (0.00)	possibly damaging (1.804)	Doolan (2005) JMCC Ingles J (2005) J Med Genet
71	TNNT2	1	g.199601032C>A	NM_001001430.1	c.291G>T	10	p.Lys97Asn	Maron B (2010) Am J Cardiol	no	pathogenic	-	Highly (1.0)	Highly	basic	polar uncharged	94	C0	affect protein function (0.00)	probably damaging (1.000)	Seidman (2001) Cardiogenomics
72	TNNT2	1	g.199594996C>T	NM_001001430.1	c.832C>T	17	p.Arg278Cys	Watkins (1995) N Engl J Med	yes	pathogenic	yes	Highly (1.0)	Weakly	basic	hydrophobic	180	C0	affect protein function (0.00)	probably damaging (1.000)	Yanaga (1999) J Biol Chem Pasquale (2011) Circ CV Gen
73	TNNI3	19	g.60359405del	NM_000363.4	c.258del	5	p.Leu88TrpfsX27	yes	no	likely pathogenic	?	Weakly (0.0)	-	-	-	-	-	-	-	
74	TNNI3	19	g.60357937C>A	NM_000363.4	c.356C>A	6	p.Thr119Asn	yes	no	likely pathogenic	?	Weakly (0.0)	Moderately	polar uncharged	polar uncharged	65	C0	affect protein function (0.00)	benign (0.000)	
75	TNNT2	1	g.199602596A>C	NM_001001430.1	c.196A>C	7	p.Lys66Gln	yes	no	likely pathogenic	yes	Highly (1.0)	Weakly	basic	polar uncharged	53	C0	tolerated (0.36)	probably damaging (0.999)	
76	TNNT2	1	g.199594996C>T	NM_001001430.1	c.832C>T	17	p.Arg278Cys	Watkins (1995) N Engl J Med	yes	pathogenic	yes	Highly (1.0)	Weakly	basic	hydrophobic	180	C0	affect protein function (0.00)	probably damaging (1.000)	Yanaga (1999) J Biol Chem Pasquale (2011) Circ CV Gen
77	TNNT2	1	g.199594996C>T	NM_001001430.1	c.832C>T	17	p.Arg278Cys	Watkins (1995) N Engl J Med	yes	pathogenic	yes	Highly (1.0)	Weakly	basic	hydrophobic	180	C0	affect protein function (0.00)	probably damaging (1.000)	Yanaga (1999) J Biol Chem Pasquale (2011) Circ CV Gen
78	TNNI3	19	g.55663249C>T	NM_000363.4	c.586G>A	8	p.Asp196Asn	Niimura H (2002) Circulation	yes	pathogenic	-	Highly (1.0)	Highly	acidic	polar uncharged	23	C0	affect protein function (0.00)	probably damaging (0.995)	Richard (2003) Circulation Mogensen(2004) JACC
79	ACTC1	15	g.35084404G>A	NM_005159.4	c.695C>T	5	p.Ala232Val	yes	no	likely pathogenic	-	Highly (1.0)	Highly	hydrophobic	hydrophobic	64	C65	affect protein function (0.00)	possibly damaging (0.911)	
80	ACTC1	15	g.35084711C>T	NM_005159.4	c.514G>A	4	p.Ala172Thr	yes	no	likely pathogenic	-	Highly (1.0)	Highly	hydrophobic	polar uncharged	58	C0	affect protein function (0.00)	probably damaging (0.964)	

Legend: Pt.ID= patient identification number; C=chromosome; Novelty=first publication of appearance, “yes” if mutation is previously unpublished; dbSNP= “yes” if variant is present in dbSNP public SNP database; Fam. Segregation= “yes” if familial segregation could be proved in at least one patient of the cohort for a given variant; N conservation= nucleotide conservation among species; AA conservation= aminoacid conservation among species; AA wt= aminoacid present in wild type protein; AA mut= aminoacid present in mutant protein; Grantham score= In silico prediction of pathogenic effect reporting the calculated Grantham Variation, GV, assessing if variation is tolerated (Invariable – conservative➔GV=0; Variable – conservative ➔ 0<GV<62; Variable – non-conservative ➔GV>62); AGVGD Class: Scoring of the variant using Align-GVGD algorithm (see http://agvgd.iarc.fr/classifiers.php for output classification); SIFT score: classification of the variant according to Sorting Intolerant from Tolerant (SIFT) algorithm (http://sift.jcvi.org/www/SIFT_help.html#SIFT_OUTPUT_SUBST); POLYPHEN score: classification of the pathogenicity of variant according to to Poliphen algorithm (http://genetics.bwh.harvard.edu/pph/); Other publications: publications involving description of patients or functional in vitro studies on the variant. All tests were performed using Alamut® software (Interactive Biosoftware, Rouen, France).

Supplementary Table 2: thick filament mutations			
N pts	Gene	Mutation	Classification
6	MYBPC3	c.3192dup (p.Lys1065GlnfsX12)	certainly pathogenic
6	MYBPC3	c.1505G>A (p.Arg502Gln)	certainly pathogenic
3	MYBPC3	c.2258dup (p.Lys754GlufsX79)	certainly pathogenic
3	MYBPC3	c.821+1G>A p.?	certainly pathogenic
3	MYBPC3	c.1624G>C (p.Glu542Gln)	certainly pathogenic
2	MYBPC3	c.1591G>C (p.Gly531Arg)	certainly pathogenic
2	MYBPC3	c.2440_2442del (p.Lys814del)	certainly pathogenic
2	MYBPC3	c.2309-2A>G p.?	certainly pathogenic
2	MYBPC3	c.2311G>A (p.Val771Met)	certainly pathogenic
2	MYBPC3	c.1468G>A (p.Gly490Arg)	certainly pathogenic
1	MYBPC3	c.772G>A (p.Glu258Lys)	certainly pathogenic
1	MYBPC3	c.2526C>G (p.Tyr842X)	certainly pathogenic
1	MYBPC3	c.3763G>A (p.Ala1255Thr)	certainly pathogenic
1	MYBPC3	c.2429G>A (p.Arg810His)	certainly pathogenic
1	MYBPC3	c.1321G>A (p.Glu441Lys)	certainly pathogenic
1	MYBPC3	c.3005G>A (p.Arg1002Gln)	certainly pathogenic
1	MYBPC3	c.1000G>A (p.Glu334Lys)	certainly pathogenic
1	MYBPC3	c.2905C>T (p.Gln969X)	certainly pathogenic
6	MYH7	c.1988G>A (p.Arg663His)	certainly pathogenic
3	MYH7	c.2167C>T (p.Arg723Cys)	certainly pathogenic
3	MYH7	c.1954A>G (p.Arg652Gly)	certainly pathogenic
3	MYH7	c.1324C>T (p.Arg442Cys)	certainly pathogenic
2	MYH7	c.1208G>A (p.Arg403Gln)	certainly pathogenic
2	MYH7	c.2779G>A (p.Glu927Lys)	certainly pathogenic
1	MYH7	c.2606G>A (p.Arg869His)	certainly pathogenic
1	MYH7	c.1987C>T (p.Arg663Cys)	certainly pathogenic
1	MYH7	c.4130C>T (p.Thr1377Met)	certainly pathogenic
1	MYH7	c.3158G>A (p.Arg1053Gln)	certainly pathogenic
1	MYH7	c.1193G>A (p.Gly398Glu)	certainly pathogenic
1	MYH7	c.1615A>C (p.Met539Leu)	certainly pathogenic
1	MYH7	c.1358G>A (p.Arg453His)	certainly pathogenic
1	MYH7	c.1816G>A (p.Val606Met)	certainly pathogenic
1	MYH7	c.2156G>A (p.Arg719Gln)	certainly pathogenic
1	MYH7	c.2389G>A (p.Ala797Thr)	certainly pathogenic
1	MYH7	c.2302G>A (p.Gly768Arg)	certainly pathogenic
1	MYH7	c.1063G>A (p.Ala355Thr)	certainly pathogenic
1	MYH7	c.2770G>A (p.Glu924Lys)	certainly pathogenic
1	MYH7	c.809A>G (p.Lys270Arg)	certainly pathogenic
1	MYH7	c.2945T>C (p.Met982Thr)	certainly pathogenic
1	MYH7	c.4377G>T (p.Lys1459Asn)	certainly pathogenic
1	MYH7	c.2080C>T (p.Arg694Cys)	certainly pathogenic
3	MYL2	c.173G>A (p.Arg58Gln)	certainly pathogenic
1	MYL2	c.484G>A (p.Gly162Arg)	certainly pathogenic
5	MYBPC3	c.2689_2698del (p.Gly897AlafsX24)	likely pathogenic
4	MYBPC3	c.1112C>G (p.Pro371Arg)	likely pathogenic
3	MYBPC3	c.1020C>G (p.Tyr340X)	likely pathogenic
3	MYBPC3	c.1174del (p.Ala392LeufsX14)	likely pathogenic

2	MYBPC3	c.3432_3435dup (p.Phe1147TrpfsX3)	likely pathogenic
2	MYBPC3	c.3767_3769del (p.Thr1256del)	likely pathogenic
1	MYBPC3	c.3551C>A (p.Thr1184Asn)	likely pathogenic
1	MYBPC3	c.3560T>G (p.Leu1187Arg)	likely pathogenic
1	MYBPC3	c.1171del (p.Asp391ThrfsX15)	likely pathogenic
1	MYBPC3	c.3106C>T (p.Arg1036Cys)	likely pathogenic
1	MYBPC3	c.1789C>T (p.Arg597Trp)	likely pathogenic
1	MYBPC3	c.2302_2308del (p.Val768ThrfsX52)	likely pathogenic
1	MYBPC3	c.913_914del (p.Phe305ProfsX27)	likely pathogenic
1	MYBPC3	c.495G>C (p.Glu165Asp)	likely pathogenic
1	MYBPC3	c.649A>G (p.Ser217Gly)	likely pathogenic
1	MYBPC3	c.1471G>A (p.Val491Met)	likely pathogenic
1	MYBPC3	c.2849C>T (p.Ala950Val)	likely pathogenic
1	MYBPC3	c.2728C>A (p.Pro910Thr)	likely pathogenic
1	MYBPC3	c.3617_3618del (p.Gly1206GlufsX35)	likely pathogenic
1	MYBPC3	c.2113dup (p.Thr705AsnfsX3)	likely pathogenic
1	MYBPC3	c.2077G>T (p.Ala693Ser)	likely pathogenic
1	MYBPC3	c.2356G>T (p.Asp786Tyr)	likely pathogenic
1	MYBPC3	c.3413G>C (p.Arg1138Pro)	likely pathogenic
1	MYBPC3	c.3811C>T (p.Arg1271X)	likely pathogenic
1	MYBPC3	c.636C>G (p.Ser212Arg)	likely pathogenic
1	MYBPC3	c.1575T>G (p.Tyr525X)	likely pathogenic
1	MYBPC3	c.1574A>C (p.Tyr525Ser)	likely pathogenic
1	MYBPC3	c.818G>A (p.Arg273His)	likely pathogenic
1	MYBPC3	c.1408C>T (p.Arg470Trp)	likely pathogenic
1	MYBPC3	c.2153del (p.Leu718ArgfsX36)	likely pathogenic
1	MYBPC3	c.2429G>T (p.Arg810Leu)	likely pathogenic
2	MYH7	c.2727C>G (p.Ile909Met)	likely pathogenic
2	MYH7	c.1801C>T (p.Leu601Phe)	likely pathogenic
2	MYH7	c.2788G>C (p.Glu930Gln)	likely pathogenic
1	MYH7	c.4363G>T (p.Glu1455X)	likely pathogenic
1	MYH7	c.2594A>G (p.Lys865Arg)	likely pathogenic
1	MYH7	c.2069T>C (p.Met690Thr)	likely pathogenic
1	MYH7	c.208A>T (p.Thr70Ser)	likely pathogenic
1	MYH7	c.2974C>A (p.Leu992Met)	likely pathogenic
1	MYH7	c.920C>A (p.Pro307His)	likely pathogenic
1	MYH7	c.950A>G (p.Glu317Gly)	likely pathogenic
1	MYH7	c.2893G>A (p.Glu965Lys)	likely pathogenic
1	MYH7	c.1820G>A (p.Gly607Asp)	likely pathogenic
1	MYH7	c.2346C>A (p.Ser782Arg)	likely pathogenic
1	MYH7	c.1871A>G (p.Tyr624Cys)	likely pathogenic
1	MYH7	c.697G>T (p.Ala233Ser)	likely pathogenic
1	MYH7	c.2346C>A (p.Ser782Arg)	likely pathogenic
1	MYH7	c.2707G>C (p.Glu903Gln)	likely pathogenic
3	MYL2	c.58A>C (p.Met20Leu)	likely pathogenic
2	MYL2	c.401A>C (p.Glu134Ala)	likely pathogenic
1	MYL2	C.304G>A (p.Ala102Thr)	likely pathogenic

**Supplementary Table 3**	**TNNT2**	**TNNI3**	
Comparison between patients with TNNT2 and TNNI3 mutations (baseline)	n=43	n=24	P Value
**Clinical/Demographic features**			
Female	16 (37%)	12 (52%)	0.439
Age at enrollment (years)	43 ± 15	47 ± 18	0.334
Age at final evaluation (years)	47 ± 16	52 ± 19	0.256
Family history of HCM	23 (53%)	10 (42%)	0.447
Family history of sudden cardiac death	17 (39%)	8 (33%)	0.793
NYHA functional class			
I	31 (72%)	14 (58%)	0.286
II	9 (21%)	7 (29%)	0.553
III/IV	3 (7%)	3 (13%)	0.659
Angina pectoris	9 (21%)	4 (17%)	0.757
Syncope	6 (14%)	6 (26%)	0.324
Symptomatic	21 (49%)	14 (61%)	0.611
Atrial fibrillation	14 (44%)	7 (44%)	1.000
Abnormal BP response to exercise	9 (22%)	5 (21%)	0.993
Non-sustained ventricular tachycardia	13 (30%)	7 (30%)	1.000
Sustained ventricular tachycardia	2 (5%)	2 (10%)	0.614
**ECG**			
T wave inversion	27 (69%)	12 (67%)	0.439
Increased Voltage (LV hypertrophy)	25 (64%)	8 (44%)	**0.043**
Inferolateral Q waves	16 (41%)	7 (39%)	0.597
LV strain / repolarization abnormalities	19 (50%)	9 (53%)	0.617
**Echocardiography**			
Left atrium (mm)	43±9	44±9	0.664
Maximum LV wall thickness (mm)	21±7	19±7	0.266
With LV wall thickness > 30mm	3 (7%)	1 (4%)	0.633
Maximal thickness site: Septum	36 (84%)	14 (59%)	**0.039**
Apex/Concentric	7 (16%)	10 (41%)	**0.038**
LV End-diastolic diameter (mm)	45±8	42±9	0.164
LV End-systolic diameter (mm)	24±6	26±8	0.250
LV Ejection fraction (%)	66±10	65±6	0.656
With LV Ejection fraction <50%	2 (5%)	1 (4%)	0.290
LVOT Gradient (mmHg)	23±38	19±25	0.645
LVOT Obstruction	10 (23%)	4 (17%)	0.674
Moderate-to-severe mitral regurgitation	4 (9%)	1 (4%)	0.647
LV filling pattern			
Normal	14 (32%)	7 (29%)	1.000
Impaired relaxation	19 (44%)	7 (29%)	0.298
Pseudo-normalized	8 (19%)	5 (21%)	1.000
Restrictive	2 (5%)	3 (12%)	0.341
Lateral E' (cm/s)	10.6±2.9	9.4±3.6	0.141
With Triphasic LV filling	10 (23%)	5 (20%)	1.000

**Supplementary Table 4**	**TNNT2**	**TNNI3**	
Comparison between patients with TNNT2 and TNNI3 mutations (outcome)	n=43	n=24	P Value
Follow up (years)	4.8 ± 2.4	4.7 ± 2.8	0.878
**Clinical Outcomes**			
HCM related death	1 (2%)	1 (4%)	1.000
Heart failure-related	0 (0%)	1 (4%)	0.358
Sudden-unexpected	1 (2%)	0 (0%)	0.452
Resuscitated cardiac arrest /appropriate ICD shocks	3 (7%)	1 (4%)	0.442
Total with malignant arrhythmias *	5 (11%)	3 (12%)	0.642
Nonfatal stroke	1 (2%)	1 (4%)	1.000
NYHA functional class at final evaluation			
I	24 (56%)	9 (37%)	0.204
II	12 (28%)	10 (42%)	0.250
III/IV	7 (16%)	5 (21%)	0.743
With progression to NYHA class III or IV	6 (13%)	4 (16%)	0.737
**Final echocardiographic evaluation**			
LV Ejection fraction (%)	60±14	58±8	0.523
With LV Ejection fraction <50%	7 (16%)	5 (21%)	0.743
LV filling pattern			
Normal	9 (21%)	5 (21%)	1.000
Impaired relaxation	12 (28%)	4 (17%)	0.379
Pseudo-normalized	18 (42%)	8 (33%)	0.604
Restrictive	4 (9%)	7 (29%)	**0.046**
Adverse remodelling (progression to LV ejection fraction <50% and/ or restrictive LV filling)	6 (14%)	5 (21%)	0.505
**Interventions**			
Implantable cardioverter-defibrillator	9 (21%)	6 (26%)	0.764
Catheter ablation for atrial fibrillation	5 (12%)	3 (12%)	1.000
Alcohol ablation or myectomy	8 (18%)	2 (8%)	0.311
**Pharmacological Therapy**			
On treatment	38 (88%)	23 (96%)	0.408
Beta-blockers	28 (66%)	17 (74%)	0.275
Verapamil	10 (23%)	5 (22%)	1.000
Amiodarone	8 (18%)	6 (26%)	0.547
Disopyramide	1 (2%)	0 (0%)	0.452
Diuretics	9 (21%)	8 (35%)	0.380
ACE-inhibitors or ARB	12 (28%)	9 (39%)	0.426
Warfarin	6 (14%)	8 (35%)	0.115

Supplementary Figure 1:
progression to NYHA Class III-IV during follow-up.

Covariates	H.R.	95,0% CI for H.R. Lower	Upper	Sig.
Thin Filament mutation	2.162	1.038	4.506	.040
Presence of obstruction	4.065	1.909	8.657	.000
Comorbidities*	.611	.288	1.296	.199
Female gender	1.027	.526	2.006	.937
History of atrial fibrillation	2.745	1.306	5.773	.008
Max wall thickness >30mm	1.725	.718	4.149	.223
Age at enrollment >50 y	2.049	.961	4.365	.063
EF<50% and/or restrictive diastole at baseline	3.198	1.396	7.328	.006

*=chronic obstructive pulmonary disease, diabetes, coronary artery disease

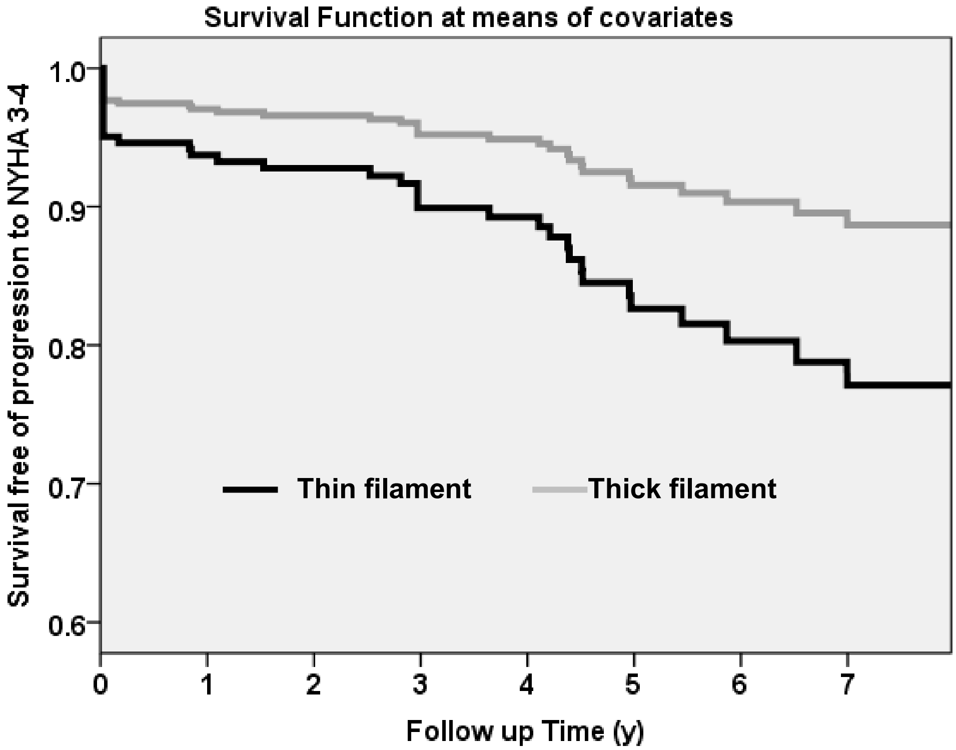

Legend. Upper panel: outcome of Cox analysis (outcome parameter: progression to NYHA Class III/IV during follow-up), showing hazard ratios (H.R.), 95% confidence intervals of H.R. and significance (Sig.) for a number of covariates evaluated at the first visit. Lower Panel: Estimated survival curves for thin and thick filament patients at means of all other covariates. Notably, the difference between the two cohorts is still clearly significant.

Supplementary Figure 2:
Adverse LV remodeling during follow-up

Covariates	H.R.	95,0% CI for H.R. Lower	Upper	Sig.
Thin Filament mutation	2.277	1.169	4.437	.016
Presence of obstruction	.475	.171	1.324	.155
Comorbidities*	1.562	.776	3.142	.211
Female gender	2.079	1.069	4.043	.031
History of atrial fibrillation	2.329	1.125	4.822	.023
Max wall thickness >30mm	.124	.016	.933	.043
Age at enrollment >50 y	1.018	.513	2.019	.959
NYHA Class 3 or 4	2.694	1.029	7.057	.044

*=chronic obstructive pulmonary disease, diabetes, coronary artery disease

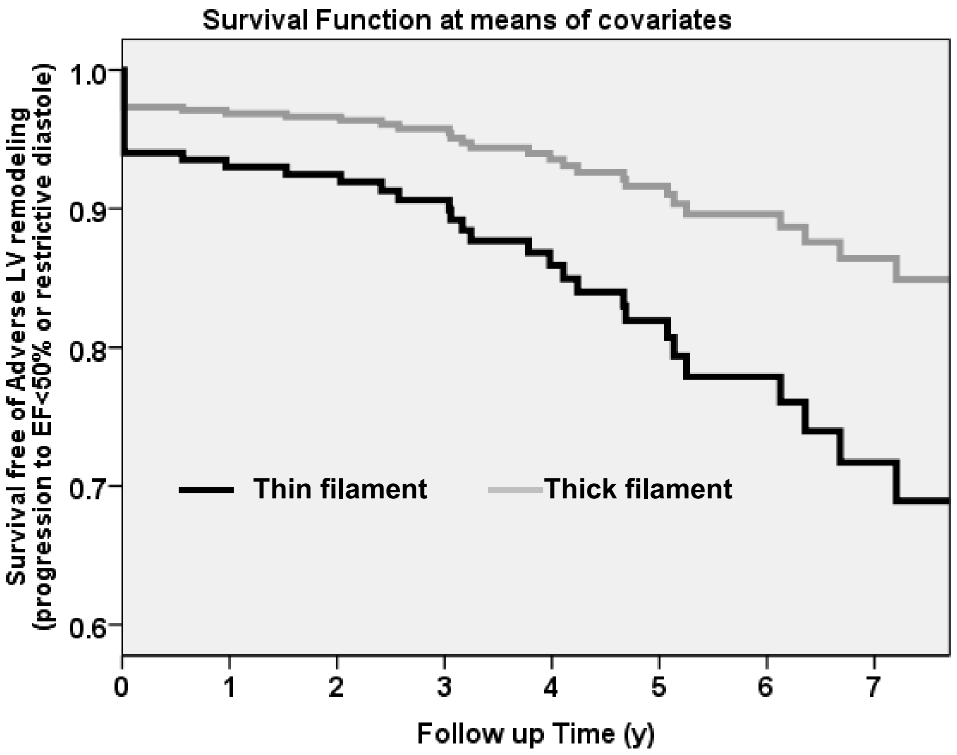

Legend. Upper panel: outcome of Cox analysis (outcome parameter: occurrence of adverse LV remodeling, i.e. progression to ejection fraction <50% or restrictive diastole, during follow-up), showing hazard ratios (H.R.), 95% confidence intervals of H.R. and significance (Sig.) for a number of covariates evaluated at the first visit. Lower Panel: Estimated survival curves for thin and thick filament patients at means of all other covariates. Notably, the difference between the two cohorts is still clearly significant.

Supplementary Figure 3:
Lifetime occurrence of Adverse LV remodeling (cross-sectional)

Covariates	H.R.	95,0% CI for H.R. Lower	95,0% CI for H.R. Upper	Sig.
Thin Filament mutation	2.792	1.483	5.257	.001
Comorbidities*	1.344	.710	2.544	.364
Female gender	1.762	.925	3.355	.085
Familial history of HCM	.870	.448	1.692	.682
Familial history of SCD	1.063	.509	2.218	.871

*=chronic obstructive pulmonary disease, diabetes, coronary artery disease

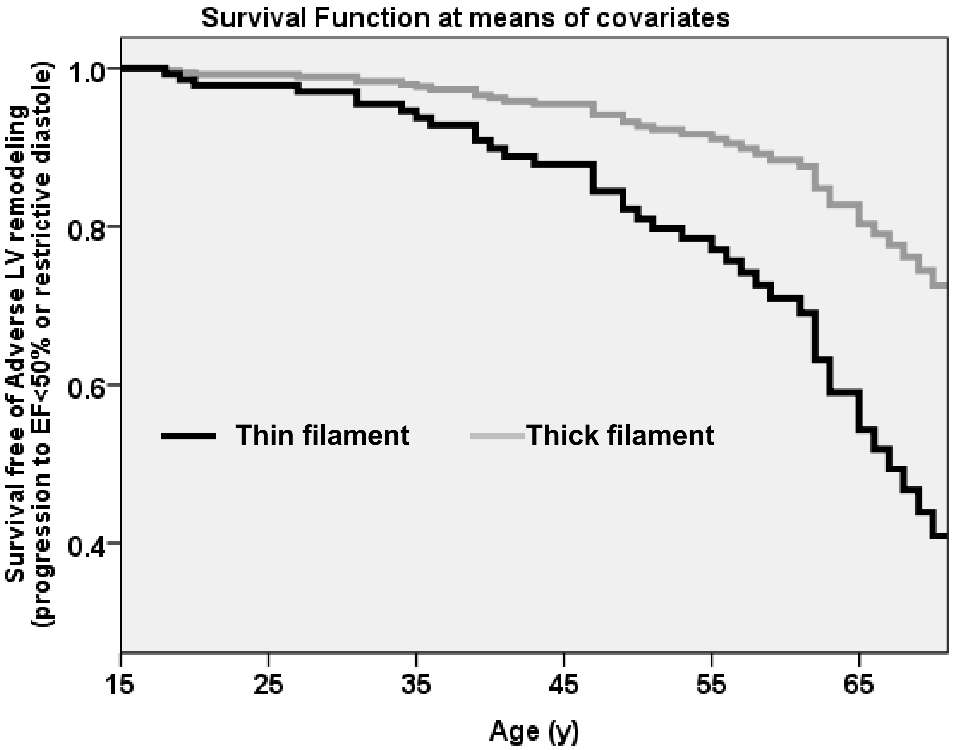

Legend. Upper panel: outcome of Cox analysis (outcome parameter: cross-sectional lifetime occurrence of LV adverse remodeling, i.e. progression to ejection fraction <50% or restrictive diastole), showing hazard ratios (H.R.), 95% confidence intervals of H.R. and significance (Sig.) for a number of covariates evaluated at the first visit. Lower Panel: Estimated survival curves for thin and thick filament patients at means of all other covariates. Notably, the difference between the two cohorts is still clearly significant.
